# Comparative chloroplast genomes of *Argentina* species: genome evolution and phylogenomic implications

**DOI:** 10.3389/fpls.2024.1349358

**Published:** 2024-04-30

**Authors:** Qin-Qin Li, Zhi-Ping Zhang, Aogan, Jun Wen

**Affiliations:** ^1^ College of Life Science and Technology, Inner Mongolia Normal University, Hohhot, China; ^2^ Key Laboratory of Biodiversity Conservation and Sustainable Utilization in Mongolian Plateau for College and University of Inner Mongolia Autonomous Region, Hohhot, China; ^3^ Department of Botany, National Museum of Natural History, Smithsonian Institution, Washington, DC, United States; ^4^ College of Computer Science and Technology, Inner Mongolia Normal University, Hohhot, China

**Keywords:** *Argentina*, Potentilleae, adaptive evolution, chloroplast genome, comparative analyses, phylogeny

## Abstract

The genus *Argentina* Hill belongs to the tribe Potentilleae Sweet and contains approximately 75 species predominantly distributed in the Sino-Himalayan region and the Malesian archipelago. So far we have less knowledge on the phylogenetic relationships within *Argentina* owing to limited sampling of *Argentina* taxa or gene fragments in previous studies. Moreover, to date there is no phylogenetic study on *Argentina* from the perspective of comparative chloroplast (cp) genomics. Here we performed comparative genomic analyses on the cp genomes of 39 accessions representing 18 taxa of *Argentina*. The *Argentina* cp genomes presented the typical quadripartite structure, with the sizes ranging from 155 096 bp to 157 166 bp. The 39 *Argentina* cp genomes contained a set of 112 unique genes, comprising four ribosomal RNA (rRNA) genes, 30 transfer RNA (tRNA) genes, as well as 78 protein-coding genes (PCGs). The cp genome organization, gene content and order in *Argentina* were highly conserved, but some visible divergences were present in IR/SC boundary regions. Ten regions (*trnH-GUG-psbA, trnG-GCC-trnfM-CAU, trnD-GUC-trnY-GUA, rpl32-trnL-UAG, atpH-atpI, rps16-trnQ-UUG, trnS-GCU-trnG-UCC, ndhF-rpl32, trnR-UCU-atpA, and accD-psaI*) were identified as excellent candidate DNA markers for future studies on species identification, population genetics and phylogeny of *Argentina*. Our results indicated that *Argentina* is monophyletic. In the current sampling, the *A. smithiana* - *A. anserina* clade was sister to the remainder of *Argentina*. Our results corroborated the previous taxonomic treatments to transfer *A. phanerophlebia* and *A. micropetala* from the genus *Sibbaldia* L. to *Argentina*. Our results showed close relationships among *A. stenophylla*, *A. microphylla*, *A. taliensis*, and *A. tatsienluensis*, congruent with previous studies based on the morphology of these species. Twenty-six genes (*rps3, rps15, rps16, rps19, rpl16, rpl20, rpl22, rpoA, rpoB, rpoC1, rpoC2, atpA, atpF, psbB, psbF, ndhA, ndhB, ndhC, ndhD, ndhF, rbcL, accD, ccsA, matK, ycf1, ycf2*) were with sites under positive selection, and adaptive evolution of these genes might have played crucial roles in *Argentina* species adaptation to the harsh mountain environment. This study will facilitate future work on taxonomy, phylogenetics, and adaptive evolution of *Argentina*.

## 1 Introduction

In green plants, the chloroplast (cp) is a unique semi-autonomous organelle (Palmer, 1991; Sato et al., 2003), playing a vital role during photosynthesis and synthesis of metabolites (Neuhaus and Emes, 2000; Rodríguez-Ezpeleta et al., 2005). The land plants cp genomes are circular form generally ranging from 115 to 165 kb and containing 120–130 genes (Ravi et al., 2008; Daniell et al., 2016), and those cp genomes are usually quadripartite in structure, with a small single-copy region (SSC) and a large single-copy region (LSC) divided by two inverted repeats (IRs) (Wicke et al., 2011; Daniell et al., 2016). Compared with mitochondrial or nuclear genomes, the cp genomes of land plants are relatively highly conserved in structure, sequence, gene content and order (Palmer, 1991; Wicke et al., 2011; Mower and Vickrey, 2018). Because of advantages of the cp genome such as characterized by usually uniparental inheritance, lack of genetic recombination, and a moderate nucleotide substitution rate (Palmer, 1985; Wolfe et al., 1987; Drouin et al., 2008; Ravi et al., 2008; Wicke et al., 2011; Mower and Vickrey, 2018), cp genomes have been the primary workhorse for researches of phylogenetics, taxonomy, evolution, and species identification in land plants (Zhang et al., 2017; Niu et al., 2018; Fu et al., 2019; Sousa et al., 2020; Daniell et al., 2021; Wu et al., 2021; Du et al., 2022; Wang et al., 2022; Zhang et al., 2022; Hu et al., 2023; Xu et al., 2023; Zhou et al., 2024).

The genus *Argentina* Hill, belonging to the tribe Potentilleae Sweet (Dobeš and Paule, 2010; Feng et al., 2015; 2017; Li et al., 2024), comprises approximately 75 species predominantly distributed in the Sino-Himalayan region and the Malesian archipelago (Li et al., 2003; Soják, 2010; Kechaykin and Shmakov, 2016). Hill (1756) first separated *Argentina* from *Potentilla* L. Rydberg (1898, 1908) supported Hill’s treatment based upon the differences in position of style; *Argentina* possesses lateral styles, while *Potentilla* has subterminal ones. Later botanists rarely accepted the genus *Argentina* and treated it as an infrageneric group of the genus *Potentilla* (Wolf, 1908; Yü and Li, 1980, 1985; Soják, 1994; Ikeda and Ohba, 1999; Li et al., 2003). Soják (1989, 1994, 2004, 2008) conducted a series of studies on the taxonomic rank and delimitations of *Argentina*. Until 2010, Soják found that the difference in stipule structure is a main morphological character that distinguishes *Argentin*a from *Potentilla*. The consistent difference is that *Argentina* has ventral stipular auricles, but *Potentilla* s. str. possesses lateral ones (Soják, 2010). Soják (2010, 2012a, b, c) recognized *Argentina* as a separate genus, according to the difference in stipule structure and results of phylogenetic studies (Eriksson et al., 2003; Dobeš and Paule, 2010). Previous phylogenetic studies (Eriksson et al., 2003; Dobeš and Paule, 2010; Feng et al., 2015, 2017; Li et al., 2024) support that *Argentina* is distinct from *Potentilla.* So far, there is no phylogenetic study on *Argentina* from the perspective of comparative chloroplast genomics. We have little knowledge on the phylogenetic relationships within *Argentina* because of the limited taxon sampling of *Argentina* or gene fragments in previous studies, which mainly concentrated on phylogenetics of Rosaceae, Rosoideae, Potentilleae, Fragariinae, *Potentilla*, or *Sibbaldia* or report of new *Argentina* species (Potter et al., 2002, 2007; Eriksson et al., 2003; Lundberg et al., 2009; Dobeš and Paule, 2010; Töpel et al., 2011; Eriksson et al., 2015; Feng et al., 2015, 2017; Persson et al., 2020; Li et al., 2024; Xue et al., 2024). Divergence time estimates indicated that the crown group of *Argentina* originated in the early Miocene (ca. 18.64 Ma), with relatively old origin compared with other genera within Potentilleae (Li et al., 2024). *Argentina* species are mostly found in the alpine or subalpine regions, sometimes as the dominant plants (Kalkman, 1989, 1993; Li et al., 2003), and the study of their cp genome adaptation to high-altitude environments is a fascinating question.

Here we conducted comparative genomic analyses on the cp genomes of 39 accessions representing 18 taxa of *Argentina* by means of bioinformatics. The objectives were to (1) analyze features of *Argentina* cp genomes, (2) screen divergence hotspots as candidate molecular markers and potential specific barcodes for *Argentina*, (3) provide insights into the phylogenetic relationships among *Argentina* species, and (4) explore the adaptive evolution of chloroplast genes of *Argentina* species. This study will contribute to further studies on species identification, population genetics, phylogenetics, and cp genome evolution of *Argentina*, and provide a theoretical basis for conservation efforts of *Argentina*.

## 2 Materials and methods

### 2.1 Taxon sampling, DNA extraction, and Illumina sequencing

In our study, a total of 18 *Argentina* taxa represented by 39 *Argentina* accessions were sampled. In addition, seven *Potentilla* species were chosen as outgroups in the phylogenomic analyses based on previous work (Feng et al., 2017; Zhang et al., 2017; Li et al., 2024). GenBank accession numbers and voucher information of the sampled taxa are presented in **Table 1**. For fresh or silica-dried leaf samples, genomic DNA was isolated by the CTAB protocol (Doyle and Doyle, 1987). For herbarium leaves sample, genomic DNA was extracted by the SDS method (Dellaporta et al., 1983; Johnson et al., 2023). The extracted DNA was sheared into fragments using sonication. These fragments were used for short-insert library construction with 300 bp insert size by NEBNext^®^ Ultra™ II DNA Library Prep Kit for Illumina^®^. Finally, the Illumina HiSeq platform in Novogene was used to sequence the pooled libraries.

**Table 1:** Summary of voucher specimens and chloroplast genome characteristics for *Argentina* species and related outgroups.

Species	Voucher	GenBank accession	Size (bp)				Number of genes				GC content (%)				References
			Total	LSC	SSC	IR	Total	Protein-coding	tRNA	rRNA	Total	LSC	SSC	IR	
*Argentina anserina* (L.) Rydb. 1	Li QQ 20180702002 (NMTC)	MW307915	155671	85066	18711	25947	129	84(6)	37(7)	8(4)	36.8	34.6	30.5	42.6	Li et al. (2024)
*Argentina anserina* (L.) Rydb. 2	Li QQ 20150806010 (NMTC)	OR863686	155096	85006	18680	25705	129	84(6)	37(7)	8(4)	36.7	34.5	30.7	42.6	This article
*Argentina anserina* (L.) Rydb. 3	Li QQ 20150822043 (NMTC)	OR863687	155103	85007	18692	25702	129	84(6)	37(7)	8(4)	36.8	34.5	30.7	42.6	This article
*Argentina cardotiana* (Hand.-Mazz.) Soják 1	Li QQ 20160818042 (NMTC)	OR863688	157014	86292	18712	26005	129	84(6)	37(7)	8(4)	37	34.8	31.1	42.7	This article
*Argentina cardotiana* (Hand.-Mazz.) Soják 2	Li QQ 20160818032 (NMTC)	OR863689	156725	86051	18704	25985	129	84(6)	37(7)	8(4)	37	34.9	31.1	42.7	This article
*Argentina cardotiana* (Hand.-Mazz.) Soják 3	Li QQ 20160818041 (NMTC)	MW307904	156798	86148	18654	25998	129	84(6)	37(7)	8(4)	37	34.9	31.1	42.7	Li et al. (2024)
*Argentina fallens* (Cardot) Soják 1	Li QQ 20160814171 (NMTC)	OR863690	155513	85220	17861	26216	129	84(6)	37(7)	8(4)	36.9	34.9	30.9	42.4	This article
*Argentina fallens* (Cardot) Soják 2	Li QQ 20170720018 (NMTC)	MW331288	155470	85192	17846	26216	129	84(6)	37(7)	8(4)	36.9	34.9	30.9	42.4	Li et al. (2024)
*Argentina festiva* (Soják) Soják 1	Li QQ 20160814063 (NMTC)	OR863691	156980	86165	18837	25989	129	84(6)	37(7)	8(4)	36.7	34.4	30.5	42.6	This article
*Argentina festiva* (Soják) Soják 2	Li QQ 20160814151 (NMTC)	MW307905	156976	86161	18837	25989	129	84(6)	37(7)	8(4)	36.7	34.4	30.5	42.6	Li et al. (2024)
*Argentina gombalana* (Hand.-Mazz.) Soják	Li QQ 20170720032 (NMTC)	MW307907	156059	85620	18489	25975	129	84(6)	37(7)	8(4)	37	34.9	31	42.7	Li et al. (2024)
*Argentina leuconota* (D. Don) Soják var. *brachyphyllaria* (Cardot) Soják 1	Li QQ 20170720020 (NMTC)	MW307902	155621	84431	17774	26708	129	84(6)	37(7)	8(4)	37	35	31.1	42.3	Li et al. (2024)
*Argentina leuconota* (D. Don) Soják var. *brachyphyllaria* (Cardot) Soják 2	Li QQ 20150802011 (NMTC)	OR863692	155621	84442	17763	26708	129	84(6)	37(7)	8(4)	37	35	31.1	42.3	This article
*Argentina leuconota* (D. Don) Soják var. *brachyphyllaria* (Cardot) Soják 3	Li QQ 20150729078 (NMTC)	OR863693	155650	84433	17769	26724	129	84(6)	37(7)	8(4)	37	35	31.1	42.3	This article
*Argentina leuconota* (D. Don) Soják var. *leuconota* 1	Li QQ 20170720030 (NMTC)	MW307908	155558	84403	17765	26695	129	84(6)	37(7)	8(4)	37.1	35	31.2	42.3	Li et al. (2024)
*Argentina leuconota* (D. Don) Soják var. *leuconota* 2	Li QQ 20160808024 (NMTC)	OR863694	155452	84234	17848	26685	129	84(6)	37(7)	8(4)	37.1	35	31	42.3	This article
*Argentina lineata* (Trevir.) Soják	Li QQ 20160807007 (NMTC)	MW307903	157166	86414	18784	25984	129	84(6)	37(7)	8(4)	36.6	34.3	30.4	42.6	Li et al. (2024)
*Argentina micropetala* (D. Don) Soják 1	Li QQ 20150802007 (NMTC)	OR863695	156584	85968	18608	26004	129	84(6)	37(7)	8(4)	37.1	34.9	31.3	42.7	This article
*Argentina micropetala* (D. Don) Soják 2	Li QQ 20160814172 (NMTC)	OR863696	156563	85949	18608	26003	129	84(6)	37(7)	8(4)	37.1	34.9	31.3	42.7	This article
*Argentina micropetala* (D. Don) Soják 3	Li QQ 20170720016 (NMTC)	MW307910	156584	85968	18608	26004	129	84(6)	37(7)	8(4)	37.1	34.9	31.1	42.7	Li et al. (2024)
*Argentina microphylla* (D. Don) Soják 1	Li QQ 20150812033 (NMTC)	MW307913	156671	85911	18786	25987	129	84(6)	37(7)	8(4)	37.1	35	31.2	42.7	Li et al. (2024)
*Argentina microphylla* (D. Don) Soják 2	Li QQ 20150812034 (NMTC)	OR863697	156542	85829	18793	25960	129	84(6)	37(7)	8(4)	37.1	35	31.2	42.8	This article
*Argentina microphylla* (D. Don) Soják 3	Li QQ 20150812032 (NMTC)	OR863698	156563	85857	18782	25962	129	84(6)	37(7)	8(4)	37.1	35	31.2	42.8	This article
*Argentina parvula* (Hook.f. ex Stapf) Soják	Hoogland R.D. & Pullen R. 5559 (US)	OR863707	156318	85495	18911	25956	129	84(6)	37(7)	8(4)	37	34.9	30.9	42.7	This article
*Argentina peduncularis* (D. Don) Soják 1	Li QQ 20160808052 (NMTC)	MW307909	155650	84444	17794	26706	129	84(6)	37(7)	8(4)	37	35	31	42.3	Li et al. (2024)
*Argentina peduncularis* (D. Don) Soják 2	Li QQ 20160808049 (NMTC)	OR863699	155650	84444	17794	26706	129	84(6)	37(7)	8(4)	37	35	31	42.3	This article
*Argentina phanerophlebia* (T. T. Yü et C. L. Li) T. Feng et H. C.Wang 1	Li QQ 20160809017 (NMTC)	MT114192	155565	85691	18452	25711	129	84(6)	37(7)	8(4)	37.1	35	31.2	42.8	Aogan et al. (2020b)
*Argentina phanerophlebia* (T. T. Yü et C. L. Li) T. Feng et H. C.Wang 2	Li QQ 20160814176 (NMTC)	OR863700	155504	85621	18461	25711	129	84(6)	37(7)	8(4)	37.1	35	31.2	42.8	This article
*Argentina polyphylla* (Wall. ex Lehm.) Soják 1	Li QQ 20160807019 (NMTC)	MW307916	156720	86070	18696	25977	129	84(6)	37(7)	8(4)	37	34.8	31.1	42.7	Li et al. (2024)
*Argentina polyphylla* (Wall. ex Lehm.) Soják 2	Li QQ 20150812060 (NMTC)	OR863701	156821	86150	18681	25995	129	84(6)	37(7)	8(4)	37	34.8	31.1	42.7	This article
*Argentina polyphylla* (Wall. ex Lehm.) Soják 3	Li QQ 20160818020 (NMTC)	OR863702	156718	86061	18731	25963	129	84(6)	37(7)	8(4)	37	34.8	31.1	42.8	This article
*Argentina smithiana* (Hand.-Mazz.) Soják	Li QQ CE0720 (NMTC)	MW307911	156753	86036	18719	25999	129	84(6)	37(7)	8(4)	37	34.9	31.1	42.7	Li et al. (2024)
*Argentina stenophylla* (Franch.) Soják 1	Li QQ 20160814003 (NMTC)	OR863703	156086	85348	18756	25991	129	84(6)	37(7)	8(4)	37.1	35	31.2	42.8	This article
*Argentina stenophylla* (Franch.) Soják 2	Li QQ 20160808054 (NMTC)	MW307912	156007	85221	18860	25963	129	84(6)	37(7)	8(4)	37.1	35	31.1	42.8	Li et al. (2024)
*Argentina taliensis* (W. W. Sm.) Soják 1	Li QQ 20160813031 (NMTC)	OR863704	156522	85874	18656	25996	129	84(6)	37(7)	8(4)	37.1	34.9	31.2	42.8	This article
*Argentina taliensis* (W. W. Sm.) Soják 2	Li QQ 20160813008 (NMTC)	MW307914	156523	85865	18666	25996	129	84(6)	37(7)	8(4)	37.1	34.9	31.2	42.8	Li et al. (2024)
*Argentina tatsienluensis* (Th. Wolf) Soják 1	Li QQ 20170720022 (NMTC)	MW307906	156785	86165	18704	25958	129	84(6)	37(7)	8(4)	37	34.9	31.1	42.8	Li et al. (2024)
*Argentina tatsienluensis* (Th. Wolf) Soják 2	Li QQ 20150802029 (NMTC)	OR863705	156505	85937	18646	25961	129	84(6)	37(7)	8(4)	37.1	34.9	31.2	42.8	This article
*Argentina tatsienluensis* (Th. Wolf) Soják 3	Li QQ 20150803003 (NMTC)	OR863706	156572	86051	18589	25966	129	84(6)	37(7)	8(4)	37.1	34.9	31.2	42.8	This article
*Potentilla ancistrifolia* Bunge	Li QQ 20170612001 (NMTC)	MW331287	156432	85716	18720	25998	129	84(6)	37(7)	8(4)	36.9	34.7	30.6	42.7	Li et al. (2024)
*Potentilla argentea* L.	Li QQ 20160712026 (NMTC)	MW338689	156207	85710	18573	25962	129	84(6)	37(7)	8(4)	36.9	34.7	30.7	42.7	Li et al. (2024)
*Potentilla articulata* Franch.	Li QQ 20150804002 (NMTC)	MW322842	155428	84613	18529	26143	129	84(6)	37(7)	8(4)	37.1	35	30.9	42.6	Li et al. (2024)
*Potentilla fragarioides* L.	Li QQ 20170518002 (NMTC)	MW331286	156350	85695	18617	26019	129	84(6)	37(7)	8(4)	36.9	34.8	30.7	42.7	Li et al. (2024)
*Potentilla osterhoutii* (A.Nelson) J.T.Howell	Annie M. Alexander, Louise Kellogg 1772 (US)	MW355416	155983	85325	18718	25970	129	84(6)	37(7)	8(4)	36.8	34.6	30.5	42.6	Li et al. (2024)
*Potentilla reptans* L.	M. Appelhans MA 756 (US)	MW348954	156718	85917	18779	26011	129	84(6)	37(7)	8(4)	36.9	34.8	30.5	42.8	Li et al. (2024)
*Potentilla suavis* Soják	Li QQ 20160814002 (NMTC)	MT114190	155044	84334	18452	26129	129	84(6)	37(7)	8(4)	37.2	35.2	31.1	42.7	Li et al. (2020a)

### 2.2 Chloroplast genome assembly and annotation

Trimmomatic v. 0.33 (Bolger et al., 2014) was utilized in order to clean adapters in raw high-throughput sequencing data. FastQC v. 0.11.8 (Andrews, 2018) was employed to evaluate the quality of the filtered paired-end reads. Then the filtered raw reads of each accession were used for assembly of the cp genome sequence by NOVOPlasty (Dierckxsens et al., 2017), with the parameters of genome range 120000–220000 and K-mer 39. In cp genome sequence assemblies of *Argentina* species, *rbcL* gene in *Argentina phanerophlebia* (GenBank accession no. MT114192) was set as the seed and its cp genome was used as the reference. Using cp genome of *Argentina phanerophlebia* (MT114192) as the reference, cp genome annotation of *Argentina* species was performed using Geneious Prime (Kearse et al., 2012) by transferring annotations. The initial annotation results were then manually checked and adjusted in Geneious Prime.

### 2.3 Chloroplast genome comparative analyses

The statistics of genome size, LSC/SSC/IR size, number of genes and GC content were summarized in Geneious Prime. With the purpose of detecting potential rearrangements and inversions, the cp genomes alignment of *Argentina* species was implemented in MAUVE v. 2.4.0 using the progressiveMauve algorithm (Darling et al., 2004, 2010). The divergence in the LSC/IR/SSC boundaries among 39 cp genomes in *Argentina* was compared and illustrated to detect the IR expansion/contraction. The mVISTA program (Frazer et al., 2004) was employed to visualize the divergence level among 39 *Argentina* cp genomes using Shuffle-LAGAN mode and with *A. anserina* (L.) Rydb. 1 as a reference. To screen divergence hotspots, we extracted the coding and noncoding regions separately in 39 *Argentina* cp genomes by “extract annotations” in Geneious Prime and furthermore aligned these homologous loci by MAFFT v. 7.450 (Katoh and Standley, 2013). Finally, the nucleotide variability (Pi) of each homologous locus was calculated in DnaSP v. 6.0 (Rozas et al., 2017).

### 2.4 Phylogenetic analyses

Phylogenetic relationships among the 18 *Argentina* taxa were inferred by maximum likelihood (ML) and Bayesian inference (BI) methods, with seven *Potentilla* species as outgroups to root the trees. A total of 46 cp genome sequences were aligned in MAFFT v. 7.450 (Katoh and Standley, 2013) with default parameters. Software trimAL v. 1.4 (Capella-Gutiérrez et al., 2009) was subsequently used to trim the alignment properly. The ML analysis was performed by RAxML v. 8.2.12 (Stamatakis, 2014), under GTRGAMMA model (option “-m GTRGAMMA”) as suggested in the manual, with analysis of rapid bootstrap and search for best-scoring ML tree (option “-f a”) and 1000 replicates bootstrap (option “-N 1000”). MrBayes v. 3.2.7a (Ronquist et al., 2012) was employed to conduct the BI analysis under best-fit model GTR+I+G as recommended by PartitionFinder2 (Lanfear et al., 2017) using the Corrected Akaike Information Criterion (AICc; Sugiura, 1978) according to Posada and Buckley (2004). Four parallel runs were performed, each run with three heated and one cold Markov chain Monte Carlo (MCMC) chains for 6000 000 generations, sampling one tree every 100 generations as well as starting from random trees. The initial 25% of the trees were regarded as burn-in and discarded. A majority-rule consensus tree was generated using the remaining trees. FigTree v. 1.4.4 (Rambaut, 2018) was finally used to visualize the phylogenetic trees.

### 2.5 Adaptive evolution analyses

To identify selection pressures on the *Argentina* cp genomes, non-synonymous (Ka), synonymous (Ks), and Ka/Ks ratios of 78 protein-coding genes (PCGs) of 39 *Argentina* accessions were calculated, with *Potentilla reptans* as the reference. Geneious Prime (Kearse et al., 2012) was employed to extract the 78 PCGs shared among the *Argentina* species and *Potentilla reptans*. The amino acids sequences and the relative nucleotide sequences were then aligned and converted into codon alignments by ParaAT v.2.0 (Zhang et al., 2012) with MAFFT as the multiple sequence aligner and with the 11th genetic code (-c 11). The KaKs_Calculator 2.0 program (Wang et al., 2010) was subsequently utilized for the analysis of Ka, Ks, and Ka/Ks ratios, with the 11th genetic code and the default model averaging (MA) method.

We also used site models in CodeML (Yang, 2007) executed in EasyCodeML (Gao et al., 2019) to detect positively selected sites of PCGs in 39 *Argentina* cp genomes. Firstly, 78 PCGs common to the *Argentina* species and *Potentilla* species were extracted in the cp genomes by Geneious Prime (Kearse et al., 2012). Each PCG was aligned according to its codons under MAFFT, followed by manually removing stop codons, and used as input for EasyCodeML. Moreover, these alignments were concatenated into a supermatrix and then the ML tree was established by RAxML v. 8.2.12 (Stamatakis, 2014) as an input tree. Likelihood ratio tests (LRTs) of M7 (beta) vs. M8 (beta and ω >1) and M8a (beta and ω = 1) vs. M8 were performed for detecting positive selection sites. If the LRTs were significant (p-values < 0.05), the Bayes empirical Bayes (BEB) (Yang et al., 2005) analysis was adopted in order to identify positively selected sites with the posterior probabilities threshold of 0.95.

## 3 Results and discussion

### 3.1 Chloroplast genome features

**Figure 1:**
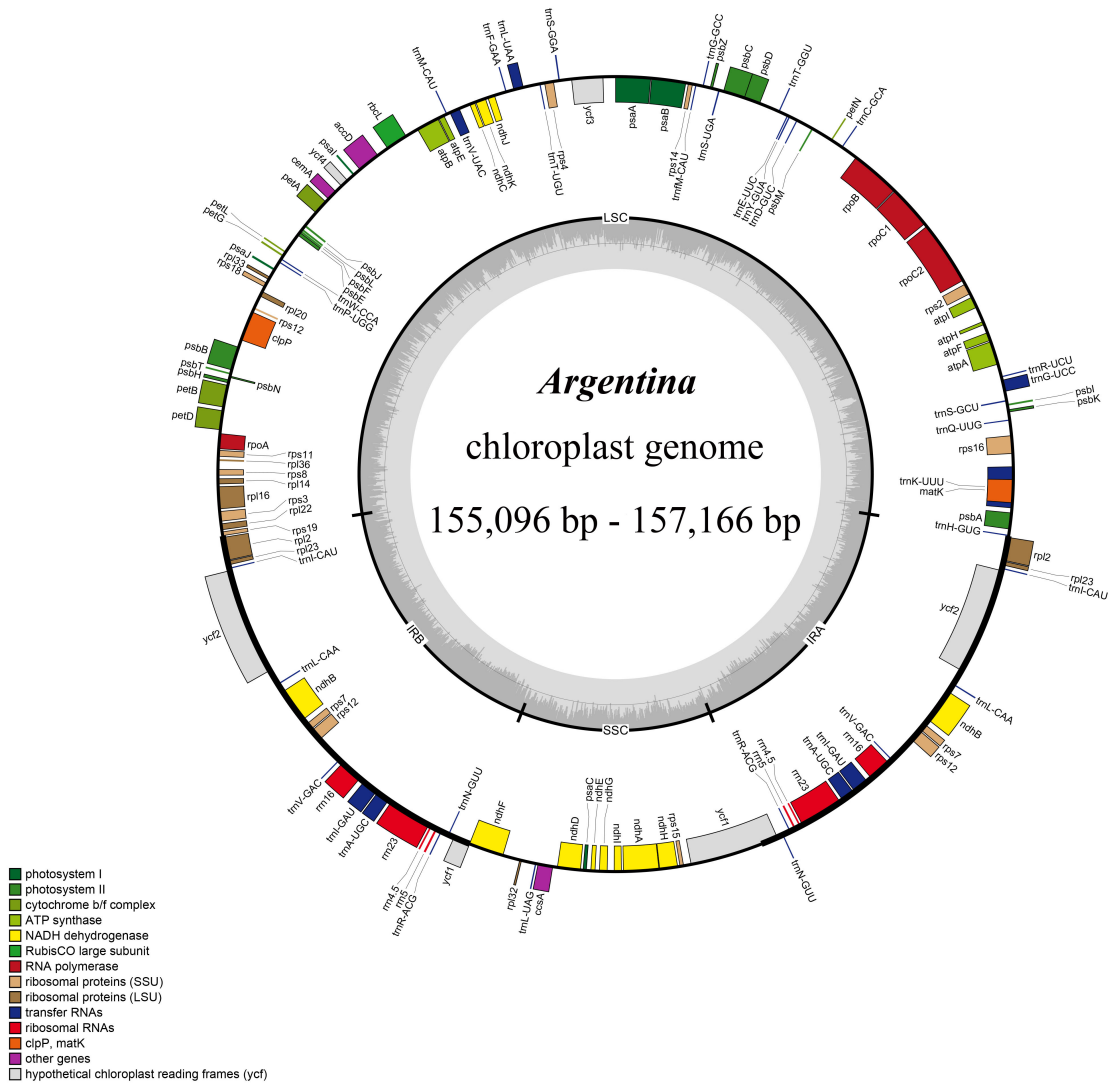
Chloroplast genome map of *Argentina* species. Genes inside the circle are transcribed clockwise, whereas those outside are transcribed counter-clockwise. The dark and light gray area in the inner circle corresponds to the GC content and AT content, respectively.

The cp genomes sizes among 39 cp genomes from 18 *Argentina* taxa ranged from 155,096 bp (*A. anserina* 2) to 157,166 bp (*A. lineata*) (**Table 1**; **Figure 1**), which was within the cp genome size range in most land plants (Ravi et al., 2008). *Argentina* cp genomes presented the typical quadripartite structure, which is consistent with that of most other land plants (Wicke et al., 2011; Daniell et al., 2016), including taxa of Rosaceae (Du et al., 2021; Li et al., 2021a; Tang et al., 2022; Wu et al., 2022; Yu et al., 2022; Zhang et al., 2023) (**Figure 1**). These cp genomes comprised two IRs separating the SSC and LSC region, with lengths of IRs from 25,702 bp (*A. anserina* 3) to 26,724 bp (*A. leuconota* var. *brachyphyllaria* 3), and with lengths of LSC and SSC from 84, 234 bp (*A. leuconota* var. *leuconota* 2) to 86,414 bp (*A. lineata*) and from 17,763 bp (*A. leuconota* var. *brachyphyllaria* 2) to 18,911 bp (*A. parvula*) respectively. The total GC content in *Argentina* cp genomes was 36.6–37.1% (**Table 1**), which is roughly comparable to that in other cp genomes of Potentilleae species (e.g., Li et al., 2020b; Rono et al., 2020; Tian et al., 2020; Zhang et al., 2020; Aogan and Li, 2020a; Li et al., 2021b). In addition, GC content in the IR region (42.3–42.8%) was higher compared with that in the SSC and LSC regions (30.4–31.3% and 34.3–35%, respectively), and this phenomenon also exists in cp genomes of other plants (e.g., Liu et al., 2021; Ogoma et al., 2022; Bai et al., 2023; Waswa et al., 2023; Zoclanclounon et al., 2023). The highest GC content in the IR region is ascribed to the existence of rRNA genes (Ravi et al., 2008).

All of the 39 *Argentina* cp genomes contained a set of 112 unique genes, comprising four ribosomal RNA (rRNA) genes, 30 transfer RNA (tRNA) genes, and 78 protein-coding genes (PCGs) (**Figure 1**; **Table 1**; **Supplementary Table S1**). Among the 112 unique genes, four rRNA genes (*rrn4.5*, *rrn5*, *rrn16*, *rrn23*), seven tRNA genes (*trnA-UGC*, *trnI-CAU*, *trnI-GAU*, *trnL-CAA*, *trnN-GUU*, *trnR-ACG*, *trnV-GAC*), and six PCGs (*ndhB*, *rpl2*, *rpl23*, *rps7*, *rps12*, *ycf2*) were duplicated. Additionally, three genes (*clpP*, *rps12, ycf3*) possessed two introns, while 14 genes (*ndhA*, *ndhB*, *petB*, *petD*, *rpl2*, *rpl16*, *rpoC1*, *rps16*, *trnA-UGC*, *trnG-UCC*, *trnI-GAU*, *trnK-UUU*, *trnL-UAA, trnV-UAC*) embraced a single intron (**Supplementary Table S1**).

**Figure 2:**
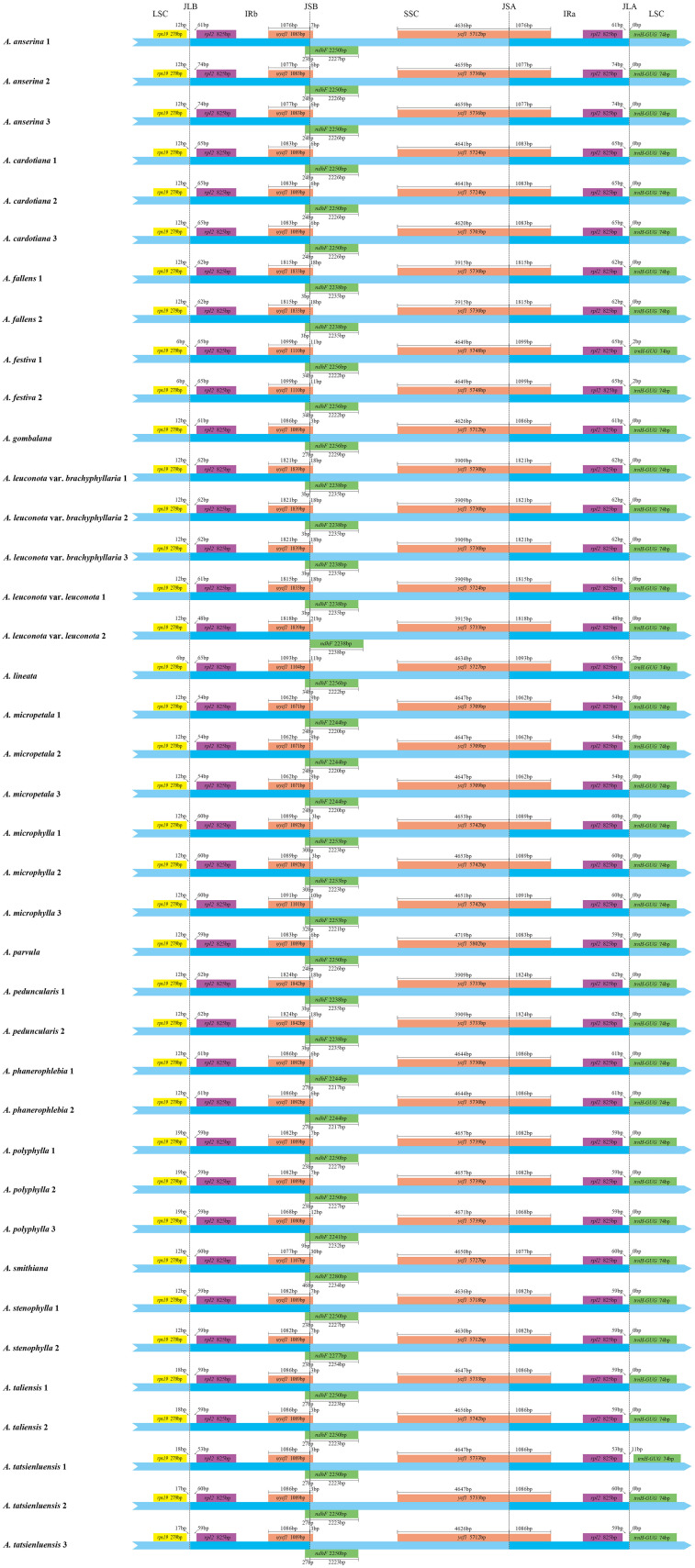
Comparison of the boundaries of the large single-copy (LSC), the small single-copy (SSC) and inverted repeat (IR) regions among 39 *Argentina* chloroplast genomes. JLB: junction line between LSC and IRb; JSB: junction line between SSC and IRb; JSA: junction line between SSC and IRa; JLA: junction line between LSC and IRa. The figure is not to scale with respect to sequence length and only shows relative changes at or near the IR/SC boundaries.

Mauve alignment analysis of 39 *Argentina* cp genomes showed no gene rearrangement and inversion and *Argentina* cp genomes had good collinearity (**Supplementary Figure S1**). Although the cp genome organization, gene content and order in *Argentina* were highly conserved, some visible divergences were present in IR/SC boundary regions (**Figure 2**). The variation of cp genome size across land plants is mainly attributed to expansion and contraction of the IR (Ravi et al., 2008; Mower and Vickrey, 2018). Comparative analysis of IR boundaries indicated that border genes were identical among *Argentina* cp genomes, but slight differences existed in lengths of these genes (*ndhF* and *ycf1*) and relative positions of these genes to the boundaries. In junctions of the LSC/IR and SSC/IR regions, the genes *rps19*-*rpl2*-*trnH* and *ycf1-ndhF* were located, with *rpl2* and *ycf1* duplicated or partially duplicated respectively in IR regions. The LSC/IRb junction (JLB) occurred between *rps19* gene and one *rpl2* gene. Gene *rps19* with 279 bp in length occurred entirely in the LSC region with 6–19 bp away from the JLB, while *rpl2* with 825 bp in length was placed completely in the IRb region, and the distance between the *rpl2* and the JLB were 48–74bp. The IRb/SSC junction (JSB) was located on the truncated *ycf1 (*ψ*ycf1*) and *ndhF.* The ψ*ycf1* pseudogene with 1071–1842 bp in length spanned the JSB boundary, with a length of 3–30 bp and 1062–1824 bp in the SSC and IRb region, separately. The length of *ndhF* was 2238–2280 bp, and the overlap between *ndhF* and ψ*ycf1* existed, in which *ndhF* expanded into the IRb region for 0–46 bp. The length of *ycf1* was 5703–5802 bp and the gene crossed over the SSC/IRa junction (JSA), with a length of 3909–4719 bp and 1062–1824 bp in the SSC and IR region. The IRa/LSC junction (JLA) was located between the other *rpl2* and *trnH-GUG*. Located in the IRa region, *rpl2* was separated from the JLA by a spacer varying from 48 bp to 74 bp. Gene *trnH-GUG* with 74bp in length was located in the LSC region, with 0–11bp away from the JLA. In general, cp genomes boundaries of *Argentina* species were greatly conserved and no obvious contraction and expansion existed in the IR region, which further supported that IR boundary shifts were relatively minor in closely related species (Zhu et al., 2016; Liu et al., 2018; Ren et al., 2022; Zhang et al., 2022). The similar findings were also observed in other genera in the tribe Potentilleae, such as *Alchemilla* (Rono et al., 2020), *Chamaerhodos* (Li et al., 2021a), and *Fragaria* (Li et al., 2021b). In addition, the boundary features of different populations of the same species were basically stable, although there are some exceptions.

The mVISTA program (Frazer et al., 2004) was employed to visualize the divergence level among 39 *Argentina* cp genomes using Shuffle-LAGAN mode and with *A. anserina* 1 as the reference. The divergence level of the 39 *Argentina* cp genomes was investigated and plotted using mVISTA (**Supplementary Figure S2**). Global comparisons of the genomic sequences revealed that at the genome-scale level, *Argentina* cp genomes were conserved, with a high degree of similarity and synteny. Additionally, the IR regions were more conserved than the SSC and LSC regions, which is a common phenomenon in most angiosperm cp genomes. Overall, compared with the coding regions, the non-coding regions were more divergent, with the highly variable non-coding regions occurred in the intergenic spacers (IGS). This finding was consistent with studies on cp genomes of other angiosperm taxa (e.g., Li et al., 2021a; Zhang et al., 2022; Bai et al., 2023).

In summary, the size, structure, GC contents, gene content and order of the *Argentina* cp genomes were highly conserved, only with slight differences in the cp genome length, GC content, and IR/SC boundary region for each species.

### 3.2 Molecular markers

**Figure 3:**
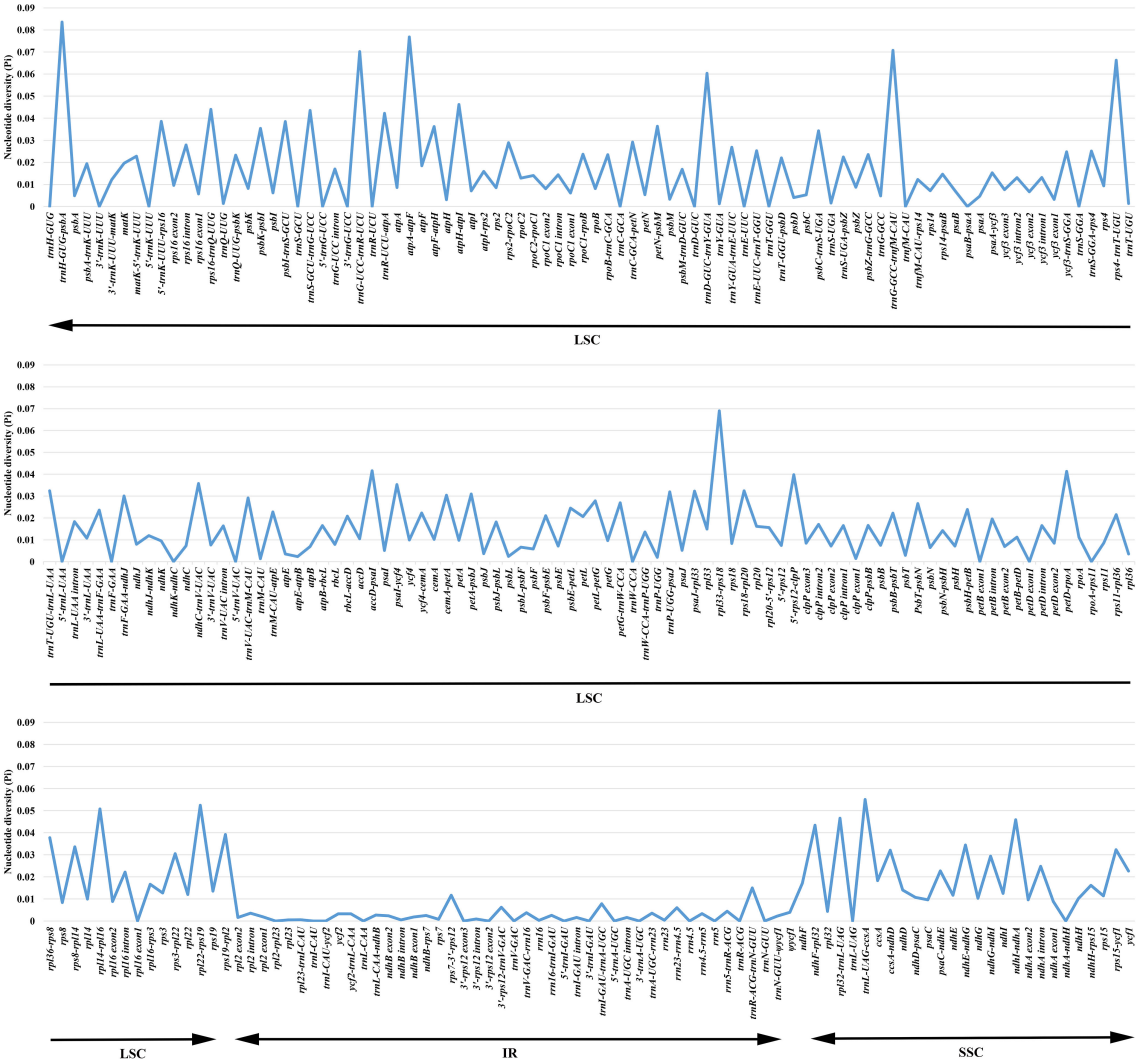
Comparison of the nucleotide diversity (Pi) values in 39 *Argentina* chloroplast genomes.

To detect the highly variable regions, the software DnaSP v. 6.0 (Rozas et al., 2017) was utilized to analyze values of the nucleotide variability (Pi) of a total of 263 exons, introns and the IGS regions of *Argentina*. The range of Pi values was 0–0.08360 and the mean value was 0.01479, which indicated *Argentina* cp genomes possessed a high level of similarity (**Supplementary Table S2**; **Figure 3**). Overall, 40 regions with Pi =0, 97 regions with 0<Pi ≤ 0.01, 51 regions with 0.01<Pi ≤ 0.02, 33 regions with 0.02<Pi ≤ 0.03, 23 regions with 0.03<Pi ≤ 0.04, and 19 regions with Pi>0.04. The results showed that regions located in the IR region had relatively low Pi values, so compared with the SSC and LSC regions, the IR region was less divergent. Moreover, compared to the coding regions, the non-coding regions were relatively more variable. Most of the highly variable regions were presented in the IGS. Nineteen regions (*trnH-GUG-psbA*, *atpA-atpF*, *trnG-GCC-trnfM-CAU*, *trnG-UCC-trnR-UCU*, *rpl33-rps18*, *rps4-trnT-UGU*, *trnD-GUC-trnY-GUA*, *trnL-UAG-ccsA*, *rpl22-rps19*, *rpl14-rpl16*, *rpl32-trnL-UAG*, *atpH-atpI*, *ndhI-ndhA*, *rps16-trnQ-UUG*, *trnS-GCU-trnG-UCC*, *ndhF-rpl32*, *trnR-UCU-atpA*, *accD-psaI*, *petD-rpoA*) with Pi>0.04 were all intergenic spacer sequences, of which four regions (*trnL-UAG-ccsA*, *rpl32-trnL-UAG*, *ndhI-ndhA*, *ndhF-rpl32*) are in the SSC region, and the other 15 regions are in the LSC region. Twenty-three regions (*5’-rps12-clpP*, *rps19-rpl2*, *5’-trnK-UUU-rps16*, *psbI-trnS-GCU*, *rpl36-rps8*, *petN-psbM*, *atpF-atpH*, *ndhC-trnV-UAC*, *psbK-psbI*, *psaI-ycf4*, *ndhE-ndhG*, *psbC-trnS-UGA*, *rps8-rpl14*, *trnT-UGU-trnL-UAA*, *rps18-rpl20*, *rps15-ycf1*, *psaJ-rpl33*, *ccsA-ndhD*, *trnP-UGG-psaJ*, *petA-psbJ*, *rps3-rpl22*, *cemA-petA*, *trnF-GAA-ndhJ*) with 0.03<Pi ≤ 0.04 were also intergenic spacer sequences; among these regions, *rps19-rpl2* was in the LSC/IR boundary, three regions (*ndhE-ndhG*, *rps15-ycf1*, *ccsA-ndhD*) occurred in the SSC region, and the remaining 19 regions were in the LSC region. The variation range of Pi values of PCGs was 0–0.02264. There were 21 PCGs (*ycf1*, *petL*, *matK*, *atpF*, *ccsA*, *ndhF*, *rpl20*, *rpl33*, *ndhD*, *rps19*, *rpoC2*, *rps3*, *ndhI*, *rpl22*, *ndhE*, *rps15*, *rpoA*, *accD*, *ndhG*, *cemA*, *ndhH*) with Pi>0.01, of which *ycf1* was in the SSC/IR boundary, *ndhF* occurred in the IR/SSC boundary, seven regions (*ccsA*, *ndhD*, *ndhI*, *ndhE*, *rps15*, *ndhG*, *ndhH*) were present in the SSC region, and the remaining 12 regions were in the LSC region.

Both the sequence variation and the sequence length should be considered when screening hypervariable regions as candidate molecular markers, because if the region length is short, it cannot provide enough informative sites. Among the 19 regions with Pi>0.04, ten regions (*trnH-GUG-psbA*, *trnG-GCC-trnfM-CAU*, *trnD-GUC-trnY-GUA*, *rpl32-trnL-UAG*, *atpH-atpI*, *rps16-trnQ-UUG*, *trnS-GCU-trnG-UCC*, *ndhF-rpl32*, *trnR-UCU-atpA*, *accD-psaI*) with suitable lengths were identified as candidate DNA markers. Among the 23 regions with 0.03<Pi ≤ 0.04, 12 regions (*5’-trnK-UUU-rps16*, *rpl36-rps8*, *petN-psbM*, *atpF-atpH*, *ndhC-trnV-UAC*, *psaI-ycf4*, *trnT-UGU-trnL-UAA*, *rps15-ycf1*, *psaJ-rpl33*, *trnP-UGG-psaJ*, *petA-psbJ*, *trnF-GAA-ndhJ*) with suitable lengths were selected as useful alternative molecular markers. Although the protein-coding genes were relatively conserved, we proposed that 15 protein-coding genes (*ycf1*, *matK*, *atpF*, *ccsA*, *ndhF*, *ndhD*, *rpoC2*, *rps3*, *ndhI*, *rpl22*, *rpoA*, *accD*, *ndhG*, *cemA*, *ndhH*) with suitable lengths could be utilized as potential DNA markers where there is a lack of information of other excellent molecular markers. Recommended by CBOL Plant Working Group (2009), *rbcL* is among the core barcodes for land plants, however, its Pi value in our study was only 0.00790. Compared with other above-mentioned molecular markers, *rbcL* has a relatively low sequence variation, so it is not suitable as a candidate molecular marker of *Argentina*. In previous studies on *Argentina* and its related taxa, chloroplast molecular markers *matK*, *ndhF*, *rbcL*, *trnC-GCA-ycf6* (*trnC-GCA-petN*), *trnL* intron, *trnL-UAA-trnF-GAA*, *trnS-trnG*, and *trnS-UGA-ycf9* (*trnS-UGA-psbZ*) were used, among which only *matK*, *ndhF*, and *trnS-trnG* belonged to the candidate molecular markers developed for *Argentina*. Thus, findings here indicated the necessity to develop exclusive molecular markers for particular groups. In general, the new candidate molecular markers developed in our study will facilitate studies on species identification, population genetics and phylogenetic studies of *Argentina*.

### 3.3 Phylogenetic implications

**Figure 4:**
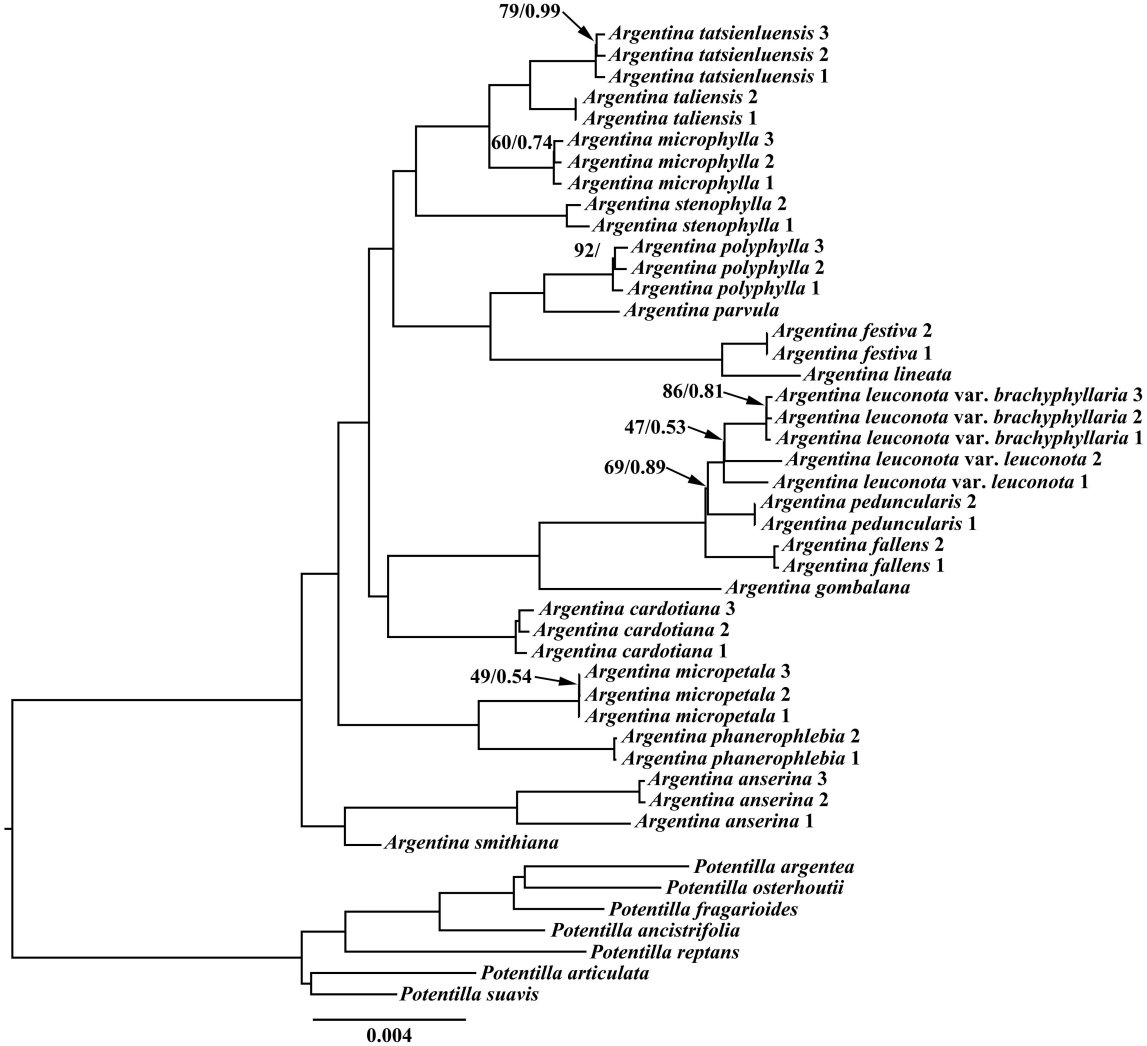
Maximum likelihood (ML) tree based on 39 complete chloroplast genome sequences from *Argentina*, with seven *Potentilla* taxa as outgroups. Values along branch represent ML bootstrap percentages (only BS < 100 % are shown) and Bayesian posterior probabilities (only PP < 1.00 are shown), respectively.

In recent years, cp genome sequences have been widely applied in plant phylogenetic studies, owning to its own merits such as containing more variable sites than the single fragment or the combination of several fragments (Wu et al., 2021; Zhang et al., 2021). In our present study, phylogenetic relationships among *Argentina* species were explored based on the cp genome sequences using ML and BI methods. The ML and BI analyses recovered identical topologies, with high maximum likelihood bootstrap support values (ML BS) and posterior probabilities (PP) across most nodes. Therefore, only the ML tree was presented in **Figure 4**. All the currently sampled *Argentina* species were clustered together with high support (**Figure 4**, ML BS = 100%, PP = 1.00). Consistent with former studies (Feng et al., 2015, 2017; Li et al., 2024), our phylogenetic results strongly supported that *Argentina* is monophyletic. Based on our current sampling, it is possible to get a glimpse into the relationships within *Argentina*. Within the sampled *Argentina* species, the clade of *A. smithiana* (Hand.-Mazz.) Soják and *A. anserina* (L.) Rydb. was sister to the remainder of the *Argentina* (**Figure 4**, ML BS = 100%, PP = 1.00). *Argentina phanerophlebia* (T.T. Yü et C. L. Li) T. Feng et H. C.Wang and *A. micropetala* (D. Don) Soják were embedded in the remaining *Argentina* species, and the result corroborated the previous taxonomic treatments to transferring those two species from the previously accepted genus *Sibbaldia* L. to *Argentina* (Soják, 2010; Feng et al., 2015)*. Argentina cardotiana* (Hand.-Mazz.) Soják was sister to a clade including *A. gombalana* (Hand.-Mazz.) Soják, *A. fallens* (Cardot) Soják, *A. peduncularis* (D. Don) Soják, *A. leuconota* (D. Don) Soják, and *A. leuconota* var. *brachyphyllaria* (Cardot) Soják (ML BS = 100%, PP = 1.00). *Argentina festiva* (Soják) Soják together with *A. lineata* (Trevir.) Soják was sister to a clade composed of *A. parvula* (Hook.f. ex Stapf) Soják and *A. polyphylla* (Wall. ex Lehm.) Soják. It is worth noting that the Malesian archipelago taxon, *A.parvula* had a close affinity to *A. festiva, A. lineata*, and *A. polyphylla.* In terms of distribution, *A. festiva* and *A. lineata* are predominantly distributed in the Sino-Himalayan region, while *A. polyphylla* occurs in the Malesian archipelago and Sino-Himalayan region. A broader sampling of *Argentina* species from the Malesian archipelago is necessary in order to decipher the phylogenetic relationships between the Sino-Himalayan taxa and the Malesian archipelago taxa in further studies. *Argentina stenophylla* (Franch.) Soják was sister to a clade comprising *A. microphylla* (D. Don) Soják, *A. taliensis* (W. W. Sm.) Soják, and *A. tatsienluensis* (Th. Wolf) Soják. The close relationship of these species indicated by our molecular phylogenetic analyses was congruent with previous studies based on the morphology of these species (Ikeda and Ohba, 1999; Li et al., 2003).

### 3.4 Adaptive evolution

Ka/Ks ratios of PCGs in *Argentina* cp genomes were less than 1 (**Supplementary Table S3**), indicating that these genes probably experienced purifying selection (Nei and Kumar, 2000). Adaptive evolution typically occurs at only a few amino acid sites, so averaging rates across all sites can result in low ability to detect positive selection (Anisimova et al., 2001). Considering that KaKs_Calculator 2.0 program (Wang et al., 2010) explores selection pressures by MA method, CodeML (Yang, 2007) implemented in EasyCodeML (Gao et al., 2019) was used to identify positively selected sites of PCGs of 39 *Argentina* cp genomes. The LRTs of M7 vs. M8 and M8a vs. M8 for PCGs of *Argentina* cp genomes were significant (p-values < 0.05), indicating that M8 (model of positive selection) should be accepted (**Table 2**). Based on BEB analysis, a total of 26 genes with sites under positive selection were detected (**Table 3**). The number of positively selected sites among these genes was 1–66: 11 genes (*atpA*, *rpoC1*, *rpoB*, *ndhC*, *psbF*, *rpl20*, *psbB*, *rpl16*, *rps19*, *ndhB*, *rps15*) possessing one site, three genes (*rpoA*, *rps3*, *ndhA*) containing two sites, five genes (*rps16*, *atpF*, *accD*, *rpl22*, *ycf2*) having three sites, two genes (*ccsA*, *ndhD*) harboring six sites, *rbcL* with 10 sites, *rpoC2* with 12 sites, two genes (*matK*, *ndhF*) with 13 sites, and *ycf1* containing the largest number of sites. These 26 genes included four small subunit of ribosome genes (*rps3*, *rps15*, *rps16*, *rps19*), three large subunit of ribosome genes (*rpl16*, *rpl20*, *rpl22*), four DNA dependent RNA polymerase genes (*rpoA*, *rpoB*, *rpoC1*, *rpoC2*), two subunits of ATP synthase genes (*atpA*, *atpF*), two subunits of photosystem II genes (*psbB, psbF*), five subunits of NADH-dehydrogenase genes (*ndhA*, *ndhB*, *ndhC*, *ndhD*, *ndhF*), subunit of Rubisco gene (*rbcL*), subunit of Acetyl-CoA-carboxylase (ACCase) gene (*accD*), c-type cytochrom synthesis gene (*ccsA*), maturase gene (*matK*), and *ycf1* and *ycf2*.

**Table 2:** Likelihood ratio tests (LRTs) statistics for detecting positive selection sites of protein-coding genes (PCGs) in *Argentina* chloroplast genomes based on site models.

Model	np	lnL	Comparison model	P-value
M8	50	-147665.580778		
M7	48	-149225.998400	M7 vs. M8	0
M8a	49	-149221.731741	M8a vs. M8	0

**Table 3:** Positively selected sites (*: P>95%; **: P>99%) identified in the chloroplast genomes of *Argentina*. Amino acids refer to sequence of *A. gombalana*.

Gene	Positively selected sites	Pr(w>1)	Number of sites
*matK*	392 G/410 L/436 L/439 N/473 I/542 I/545 P/546 K/579 Q/596 R/708 L/828 P/841 R	0.954*/0.991**/0.973*/0.993**/0.999**/0.984*/0.973*/0.997**/0.974*/0.982*/0.987*/0.991**/0.995**/	13
*rps16*	930 R/938 S/948 S	0.992**/0.955*/0.994**	3
*atpA*	1169 S	0.993**	1
*atpF*	1628 E/1642 R/1671 S	0.970*/0.981*/0.952*	3
*rpoC2*	2665 R/2841 G/3017 R/3085 A/3096 Q/3685 S/3687 D/3688 L/3690 T/3693 P/3694 K/3695 S	0.951*/0.953*/0.972*/0.970*/0.977*/1.000**/1.000**/1.000**/0.973*/0.999**/1.000**/1.000**	12
*rpoC1*	3713 H	0.972*	1
*rpoB*	4956 P	0.981*	1
*ndhC*	8763 F	0.972*	1
*rbcL*	9511 E/9569 H/9578 S/9625 P/9709 Y/9734 I/9762 S/9811 S/9926 E/9932 C	0.992**/0.992**/1.000**/0.989*/0.987*/0.993**/0.994**/0.974*/0.952*/0.953*	10
*accD*	9962 R/10028 R/10106 Q	0.995**/0.990*/0.981*	3
*psbF*	11333 F	0.999**	1
*rpl20*	11791 Q	1.000**	1
*psbB*	12650 R	0.955*	1
*rpoA*	13257 A/13449 T	0.968*/0.952*	2
*rpl16*	13971 R	0.959*	1
*rps3*	14174 I/14298 D	0.970*/0.971*	2
*rpl22*	14433 L/14434 E/14446 Q	0.983*/0.989*/0.983*	3
*rps19*	14457 K	0.958*	1
*ycf2*	16206 L/16207 P/16694 Q	0.999**/0.965*/0.959*	3
*ndhB*	17345 P	0.992**	1
*ndhF*	17907 I/17922 V/18041 L/18060 Q/18071 S/18360 G/18510 K/18518 L/18538 F/18604 L/18606 L/18611-/18614 K	0.996**/0.978*/0.961*/0.978*/0.954*/0.994**/0.992**/1.000**/0.996**/0.975*/0.994**/0.982*/1.000**	13
*ccsA*	18781 F/18784 H/18838 S/18863 I/18876 P/18894 N	0.995**/0.999**/0.978*/0.995**/0.971*/0.992**	6
*ndhD*	19057 I/19058 C/19061 I/19520 L/19521-/19526-	0.985*/0.952*/0.986*/0.999**/0.968*/0.980*	6
*ndhA*	20075 S/20171 V	0.952*/0.955*	2
*rps15*	20821 S	0.993**	1
*ycf1*	21219 G/21379 N/21433 V/21488 L/21490 P/21527 A/21541 K/21543 I/21554 P/21575 R/21587 Q/21591 N/21686 S/21694 S/21785 T/21888 E/21898 L/21905 L/21921 R/21951 M/21960 G/21984 P/22046 G/22055 R/22139 K/22148 C/22155 K/22173 T/22182 S/22186 S/22196 G/22212 L/22222 I/22224 Q/22265 H/22278 R/22314 G/22323 Q/22333 I/22393 R/22423 E/22437 Y/22444 K/22459 K/22462 I/22471 Q/22473 M/22481 Q/22482 N/22495 D/22499 K/22501 G/22502 F/22513 L/22526 P/22577 P/22585 F/22624 Q/22686 K/22744 I/22752 I/22776 H/22809 R/22811 L/22845 N/22880 G	0.967*/0.970*/0.989*/0.967*/1.000**/0.952*/ 0.987*/0.973*/0.966*/0.991**/0.977*/0.984*/ 0.980*/0.960*/0.977*/0.997**/0.974*/0.975*/ 0.967*/0.985*/0.962*/0.987*/0.962*/1.000**/ 0.999**/0.997**/0.993**/0.986*/0.984*/0.999**/ 0.974*/0.958*/0.994**/0.964*/0.997**/0.970*/ 0.975*/0.987*/0.999**/0.994**/0.964*/0.996**/ 0.989*/0.979*/0.999**/0.965*/0.986*/0.965*/ 1.000**/0.996**/0.964*/1.000**/0.993**/ 0.965*/0.981*/0.966*/0.965*/1.000**/0.965*/ 0.996**/0.978*/1.000**/0.969*/0.972*/0.992**/ 0.987*	66

The majority of *Argentina *species occurs at (sub)alpine areas above 3000 meters altitude (Kalkman, 1989, 1993; Ikeda and Ohba, 1999; Li et al., 2003; Kechaykin and Shmakov, 2016). High-altitude mountains habitats are characterized by low water availability, intense ultraviolet radiation, strong wind and/or abrasion, low air density, extreme temperatures or large diurnal/seasonal thermal fluctuations (Körner, 2003; Körner et al., 2011). The occupation of the high-altitude mountain environments indicates that *Argentina *species inhabiting these areas have adapted to conditions of high-altitude habitats. Species of *Argentina *show several morphological adaptations to high-altitude mountains habitats, such as small and compact rosettes, pinnate leaves with small leaflets, plant surface covered with hairs (Kalkman, 1989, 1993 *;* Ikeda and Ohba, 1999; Li et al., 2003). Here possible evidence of positive selection in chloroplast coding genes was detected to reveal the adaptation of *Argentina* species to high-altitude mountains habitats at the molecular level. A total of 26 genes with sites under positive selection were detected and adaptive evolution of these genes might have helped *Argentina* species to adapt to the harsh mountain environment. As important constituents of protein synthesis machinery, cp ribosomal proteins participate in various processes of plant growth, development as well as reaction to unfavorable conditions (Schippers and Mueller-Roeber, 2010; Fleischmann et al., 2011; Tiller et al., 2012; Tiller and Bock, 2014; Zhang et al., 2016; Robles and Quesada, 2022). Adaptive evolution of these seven subunits of ribosome genes *(rps3*, *rps15*, *rps16*, *rps19*, *rpl16*, *rpl20*, *rpl22*) may be helpful for the normal growth and development of *Argentina* species under the extreme environments. Previous studies revealed that 30S ribosomal protein S15 is essential for the maintenance of high cp translational capacity under the cold stress (Fleischmann et al., 2011). Four enzymatic subunits α, β, β’ and β’’ encoded by *rpoA*, *rpoB*, *rpoC1* and *rpoC2* respectively, constitute catalytic core of the plastid-encoded plastid RNA polymerase (PEP) (Zhelyazkova et al., 2012). Genes of photosystems I and II are only transcribed by PEP promoters, and PEP represents the main transcription machinery of mature chloroplasts (Hajdukiewicz et al., 1997; Zhelyazkova et al., 2012; Kindgren and Strand, 2015). Genes *rpoA*, *rpoB*, *rpoC1*, *rpoC2* were all under positive selection, which may be conducive to the transcription of photosynthetic genes of *Argentina* species in the harsh environments. Generating ATP from ADP using the proton gradient across the membrane (Hahn et al., 2018), the cp ATP synthase is essential for photosynthesis and plant growth (Yamamoto et al., 2023). Six ATP synthase subunit genes (*atpA*, *atpB*, *atpE*, *atpF*, *atpH*, and *atpI*) are encoded by the cp (Wicke et al., 2011), and two out of six genes (*atpA* and *atpF*) were subjected to positive selection in the present study. The 47 kDa chlorophyll α-binding protein (CP47) encoded by *psbB*, acts as the light-capturing antenna for the core complex of photosystem II (PSII) (Hird et al., 1991; Swiatek et al., 2003). The beta subunit of Cytochrome (cyt) b-559 protein encoded by *psbF*, is essential for proper assembly and activity of PSII reaction center (Pakrasi et al., 1990, 1991 *;* Swiatek et al., 2003 *;* Nakamura et al., 2019 *).* The positive selection pressure on *psbB* and *psbF* may reflect adaptation of PSII to the alpine environments such as strong light. The *ndh* genes (*ndhA*, *ndhB*, *ndhC*, *ndhD*, *ndhE*, *ndhF*, *ndhG*, *ndhH*, *ndhI*, *ndhJ*, *ndhK*) in cp encode eleven NADH-dehydrogenase subunits of the Ndh1-complex bound to the thylakoid membrane (Ifuku et al., 2011). The thylakoid Ndh1-complex is involved in cyclic electron transfer around photosystem I (PSI) and in chlororespiration (Suorsa et al., 2009), which seems to be important in adaptation to stress conditions, such as high light and low temperature (Endo et al., 1999; Rumeau et al., 2007; Yamori et al., 2011). Positive selection of five *ndh* genes (*ndhA*, *ndhB*, *ndhC*, *ndhD*, *ndhF*) in *Argentina* may reflect the adaptation to alpine environmental stress, such as low temperature and intense light. The *rbcL* gene encodes the Rubisco large subunit (Wicke et al., 2011). Rubisco catalyzes the assimilation of atmospheric CO2 during photosynthesis (Wilson and Hayer-Hartl, 2018; Whitney and Sharwood, 2021). In land plants, positive selection of *rbcL* is quite common (Kapralov and Filatov, 2007). Positive selection of *rbcL* in *Argentina* is related to adaptation to low CO2 concentrations in the alpine environments. Gene *accD* encodes the beta carboxyl transferase subunit of ACCase, and ACCase is an essential enzyme that catalyzes *de novo* fatty acid biosynthesis (Rawsthorne, 2002; Kode et al., 2005). The *accD* gene could affect several biological processes such as cp division, leaf development, and seed development and storage compound metabolism (Madoka et al., 2002; Kode et al., 2005; Caroca et al., 2021). The gene *ccsA *encodes cytochrome c biogenesis protein, which is essential during c-type cytochromes biogenesis in the heme attachment step (Xie and Merchant, 1996). Maturase K protein encoded by the gene *matK*, is the only putative group II intron maturase of the cp (Neuhaus and Link, 1987). The maturase *matK* is required for splicing its own and other additional group II introns (Zoschke et al., 2010) and functions in photosynthesis and plant development (Barthet and Hilu, 2007). Essential genes *ycf1* and *ycf2* in higher plant cp genomes encode products that are indispensable for cell survival (Drescher et al., 2000). In all, the 26 genes with sites under positive selection are associated with biological processes such as self-replication, photosynthesis and biosynthesis, which may have played crucial roles in *Argentina* adaptation to the harsh mountain environment.

## 4 Conclusion

In summary, comparative analyses were conducted on 39 *Argentina* cp genomes, which revealed that the size, structure, GC contents, gene content and order of *Argentina* cp genomes were highly conserved. Twenty-two regions (*trnH-GUG-psbA*, *trnG-GCC-trnfM-CAU*, *trnD-GUC-trnY-GUA*, *rpl32-trnL-UAG*, *atpH-atpI*, *rps16-trnQ-UUG*, *trnS-GCU-trnG-UCC*, *ndhF-rpl32*, *trnR-UCU-atpA*, *accD-psaI*, *5’-trnK-UUU-rps16*, *rpl36-rps8*, *petN-psbM*, *atpF-atpH*, *ndhC-trnV-UAC*, *psaI-ycf4*, *trnT-UGU-trnL-UAA*, *rps15-ycf1*, *psaJ-rpl33*, *trnP-UGG-psaJ*, *petA-psbJ*, *trnF-GAA-ndhJ*) were identified as candidate molecular markers for species identification, population genetics as well as phylogenetic researches of *Argentina*. The phylogenetic relationships among *Argentina* species were explored using the cp genome sequences, which were helpful for deciphering the evolutionary relationships of *Argentina* species. Twenty-six genes were with sites under positive selection and adaptive evolution of these genes might have helped *Argentina* species to adapt to the harsh mountain environment. Our findings provide insights into species identification, cp genome evolution and phylogeny in *Argentina*, and adaptation of *Argentina* species to high-altitude mountain habitats. Expanding species sampling and incorporating single-copy nuclear genes in further studies will contribute to deeper understanding about *Argentina* taxonomy, phylogeny, and adaptive evolution.

## Data availability statement

The datasets presented in this study can be found in online repositories. The names of the repository/repositories and accession number(s) can be found below: GenBank of NCBI (https://www.ncbi.nlm.nih.gov/genbank/), MT114190, MT114192, MW307902-MW307916, MW322842, MW331286-MW331288, MW338689, MW348954, MW355416, and OR863686-OR863707.

## Author contributions

Q-QL: Conceptualization, Formal analysis, Investigation, Writing – original draft. Z-PZ: Formal analysis, Writing – original draft. A: Investigation, Writing – original draft. JW: Conceptualization, Writing – review & editing.
