# Supplementary material for: Comparative chloroplast genomes of Argentina species: genome evolution and phylogenomic implications
Source: Front Plant Sci. 2024 Apr 30;15:1349358. doi: 10.3389/fpls.2024.1349358 (PMC11099909; doi:10.3389/fpls.2024.1349358)
Supplement: Supplementary file 1 [file DataSheet_1.zip › Supplementary Material/Captions for the Supplementary Material files.docx]

Supplementary Material

Comparative chloroplast genomes of *Argentina* species: genome evolution and phylogenomic implications

Qin-Qin Li, Zhi-Ping Zhang, Aogan, Jun Wen*

*** Correspondence:** Jun Wen: wenj@si.edu

**Supplementary Figure 1.** MAUVE alignments of 39 *Argentina* chloroplast genomes.

**Supplementary Figure 2.** mVISTA identity plot based on Shuffle-LAGAN alignment of 39 *Argentina* chloroplast genomes, with *A. anserina* 1 as a reference.

**Supplementary Table 1.** Genes contained in the *Argentina* chloroplast genome.

**Supplementary Table 2.** The nucleotide diversity (Pi) values of 263 homologous loci.

**Supplementary Table 3.** The rates of Ka, Ks and Ka/Ks of 78 genes among 39 *Argentina* accessions.
