# Supplementary material for: Comparative chloroplast genomes of Argentina species: genome evolution and phylogenomic implications
Source: Front Plant Sci. 2024 Apr 30;15:1349358. doi: 10.3389/fpls.2024.1349358 (PMC11099909; doi:10.3389/fpls.2024.1349358)
Supplement: Supplementary file 1 [file DataSheet_1.zip › Supplementary Material/Table S1.docx]

Table S1. Genes contained in the *Argentina* chloroplast genome.

| Category of genes | Group of genes | Name of genes |
| --- | --- | --- |
| Self-replication | Ribosomal RNAs | *rrn4.5*^a^*, rrn5*^a^*, rrn16*^a^, *rrn23*^a^ |
|  | Transfer RNAs | *trnA-UGC*^a#^, *trnC-GCA*, *trnD-GUC*, *trnE-UUC*, *trnF-GAA*, *trnfM-CAU*, *trnG-GCC*, *trnG-UCC*^#^, *trnH-GUG*, *trnI-CAU*^a^, *trnI-GAU*^a#^, *trnK-UUU*^#^, *trnL-CAA*^a^, *trnL-UAA*^#^, *trnL-UAG*, *trnM-CAU*, *trnN-GUU*^a^, *trnP-UGG*, *trnQ-UUG*, *trnR-ACG*^a^, *trnR-UCU*, *trnS-GCU*, *trnS-GGA*, *trnS-UGA*, *trnT-GGU*, *trnT-UGU*, *trnV-GAC*^a^, *trnV-UAC*^#^, *trnW-CCA*, *trnY-GUA* |
|  | Small subunit of ribosome | *rps2*, *rps3*, *rps4*, *rps7*^a^, *rps8*, *rps11*, *rps12*^ab##^, *rps14*, *rps15*, *rps16*^#^, *rps18*, *rps19* |
|  | Large subunit of ribosome | *rpl2*^a#^, *rpl14*, *rpl16*^#^, *rpl20*, *rpl22*, *rpl23*^a^, *rpl32*, *rpl33*, *rpl36* |
|  | DNA dependent RNA polymerase | *rpoA*, *rpoB*, *rpoC1*^#^, *rpoC2* |
| Photosynthesis | Subunits of ATP synthase | *atpA*, *atpB*, *atpE*, *atpF*, *atpH*, *atpI* |
|  | Subunits of photosystem I | *psaA*, *psaB*, *psaC*, *psaI*, *psaJ*, *ycf3*^##^, *ycf4* |
|  | Subunits of photosystem II | *psbA*, *psbB*, *psbC*, *psbD*, *psbE*, *psbF*, *psbH*, *psbI*, *psbJ*, *psbK*, *psbL*, *psbM*, *psbN*, *psbT*, *psbZ* |
|  | Subunits of cytochrome b/f complex | *petA*, *petB*^#^, *petD*^#^, *petG*, *petL*, *petN* |
|  | Subunits of NADH-dehydrogenase | *ndhA*^#^, *ndhB*^a#^, *ndhC*, *ndhD*, *ndhE*, *ndhF*, *ndhG*, *ndhH*, *ndhI*, *ndhJ*, *ndhK* |
|  | Subunit of Rubisco | *rbcL* |
| Other genes | Subunit of Acetyl-CoA-carboxylase | *accD* |
|  | C-type cytochrom synthesis | *ccsA* |
|  | Envelop membrane protein | *cemA* |
|  | Protease | *clpP*^##^ |
|  | Maturase | *matK* |
| Unkown function | Conserved open reading frame | *ycf1*, *ycf2*^a^ |

^a^ Two gene copies in IRs; ^b^ gene divided into two independent transcription units; ^#^ genes containing one intron; ^##^ genes containing two introns.
