# Supplementary figures and images for: Comparative chloroplast genomes of Argentina species: genome evolution and phylogenomic implications
Source: Front Plant Sci. 2024 Apr 30;15:1349358. doi: 10.3389/fpls.2024.1349358 (PMC11099909; doi:10.3389/fpls.2024.1349358)

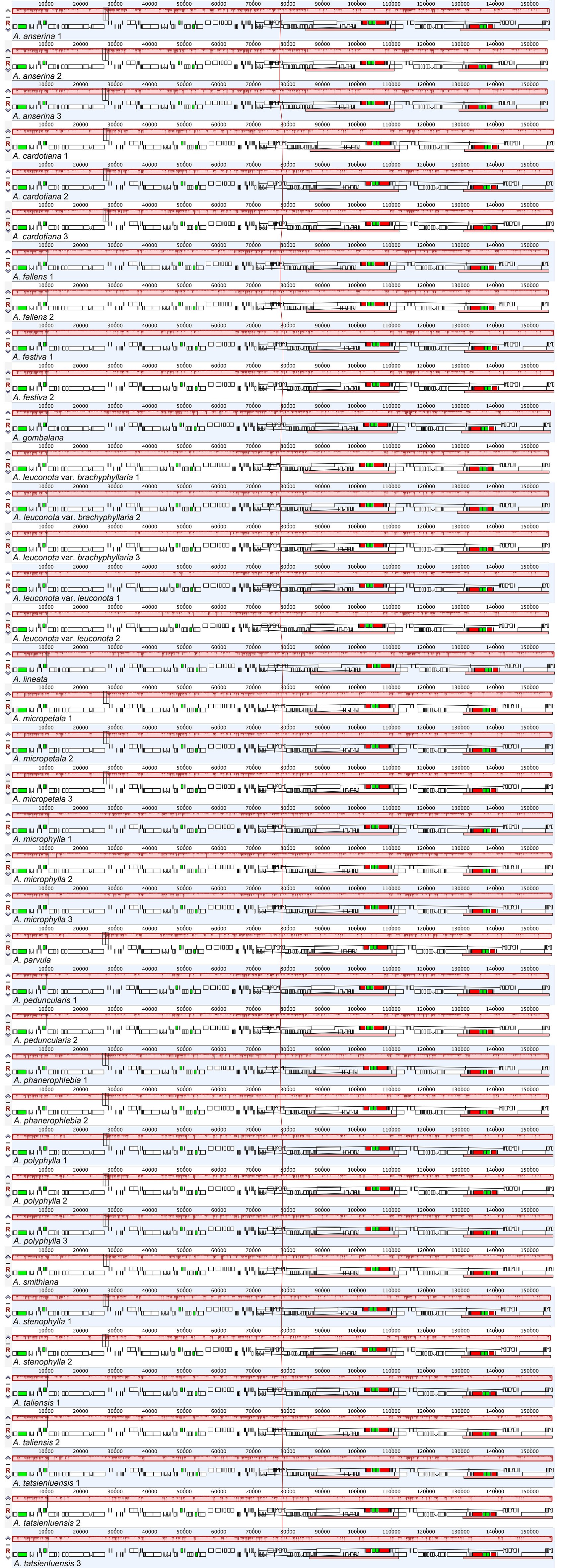

Supplement: Supplementary file 1 [file DataSheet_1.zip › Supplementary Material/Figure S1.jpg]

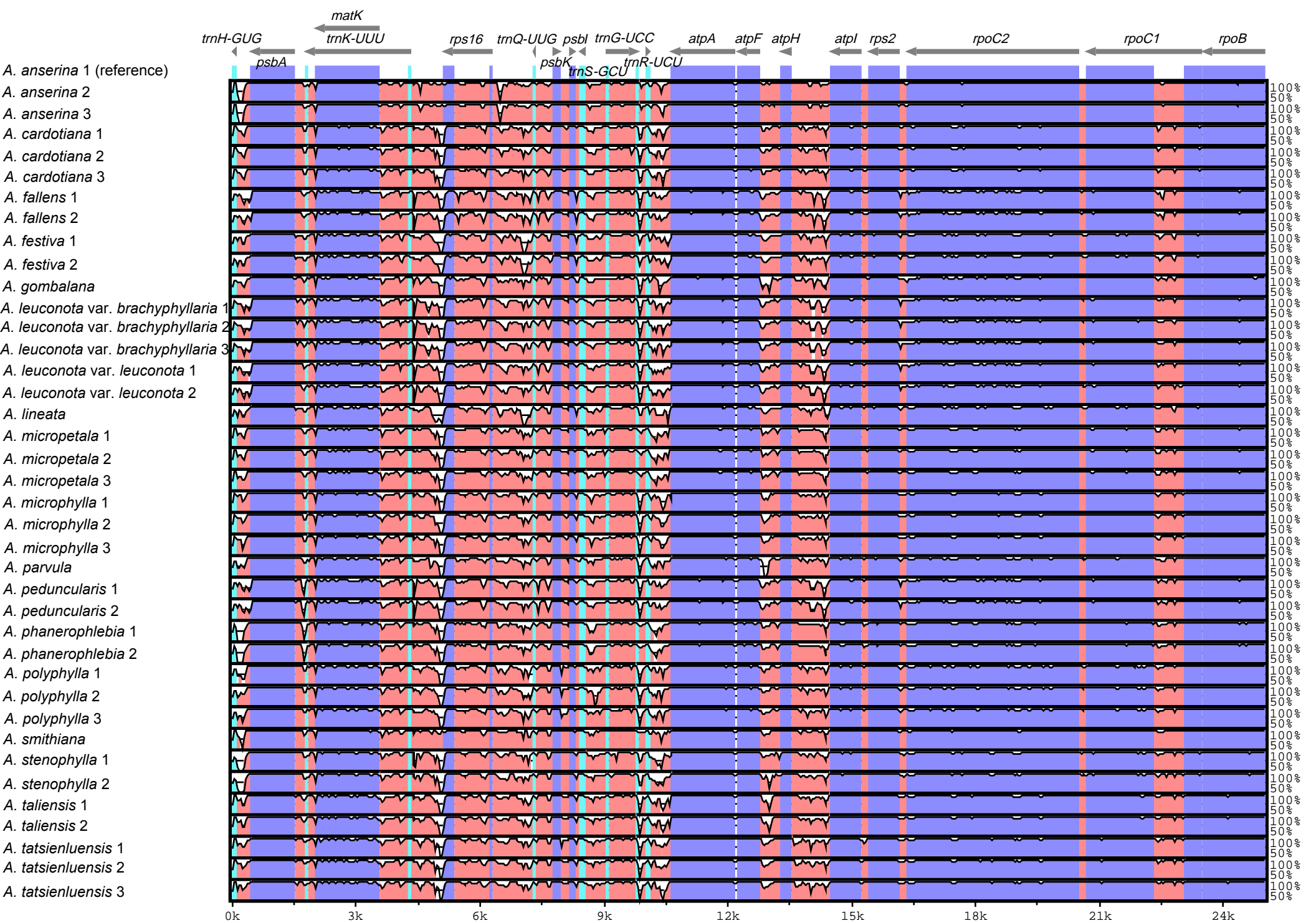

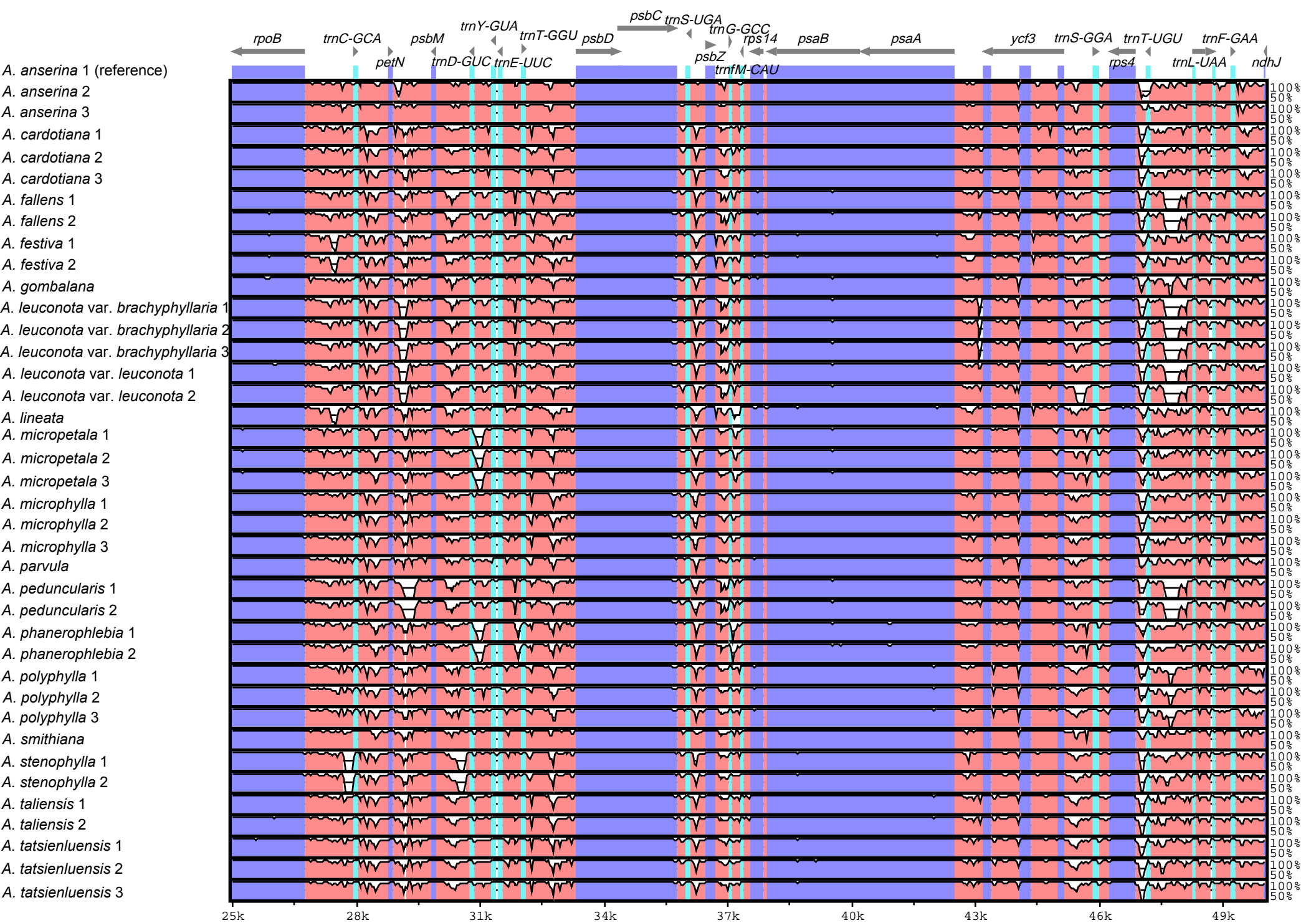

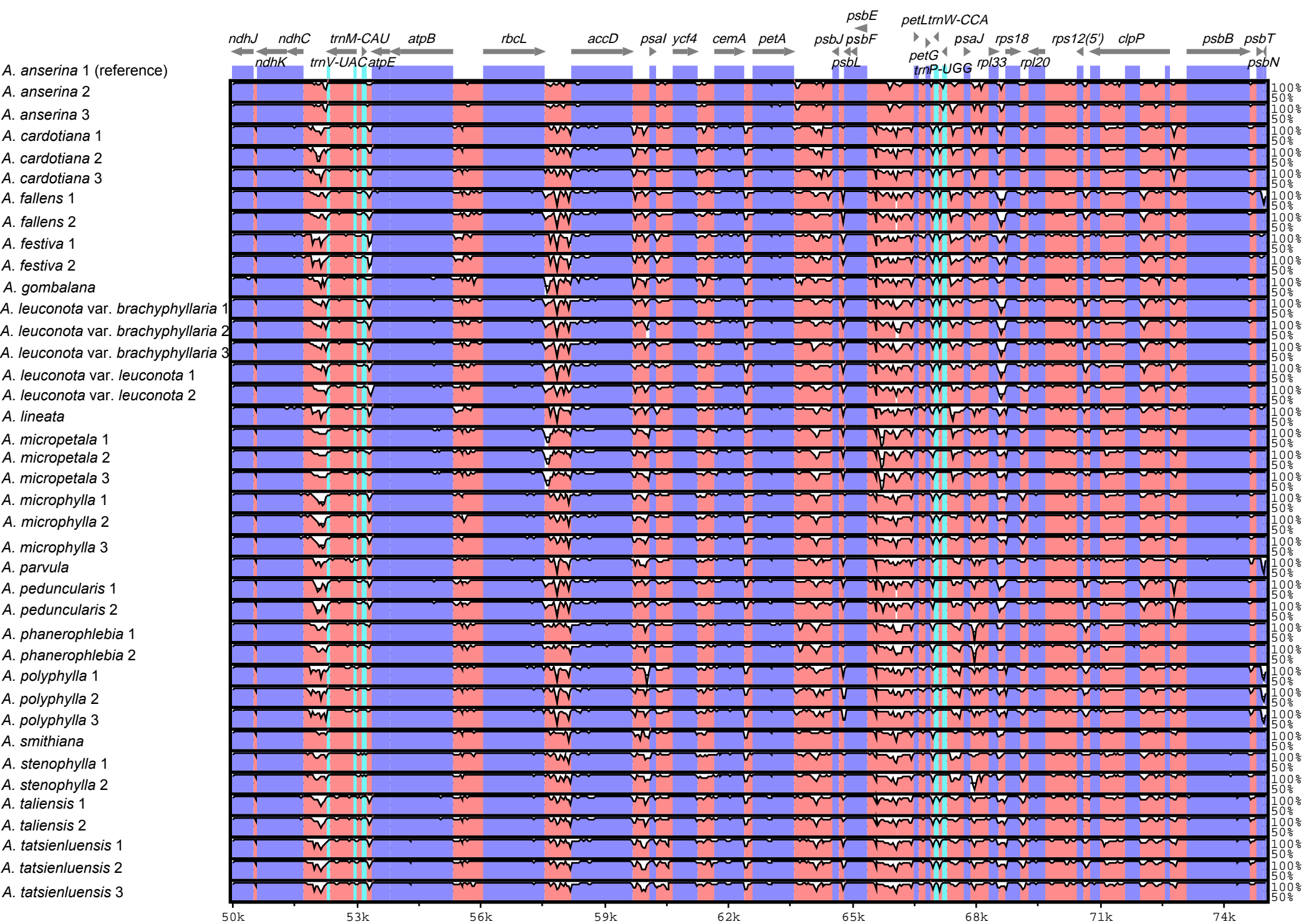

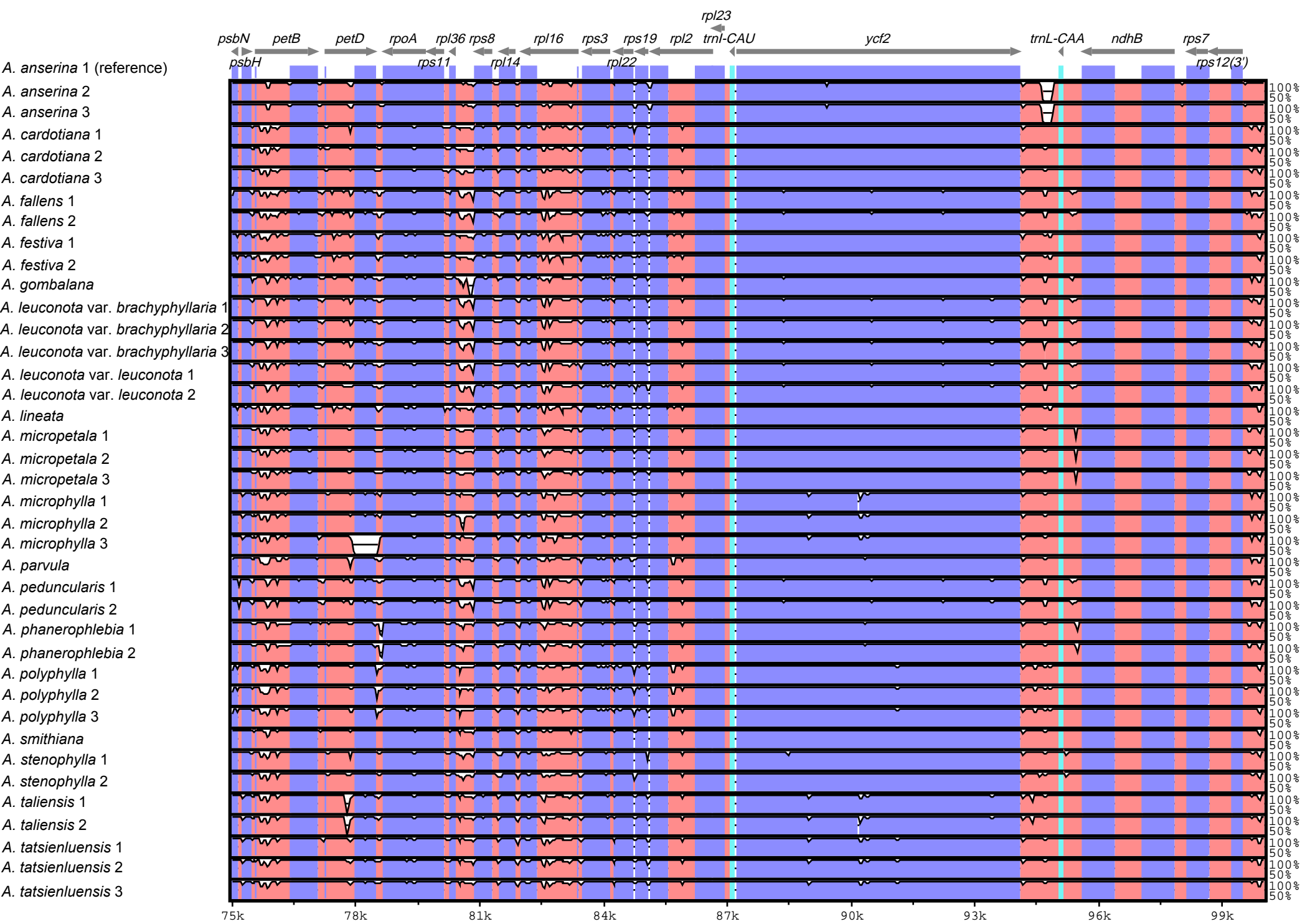

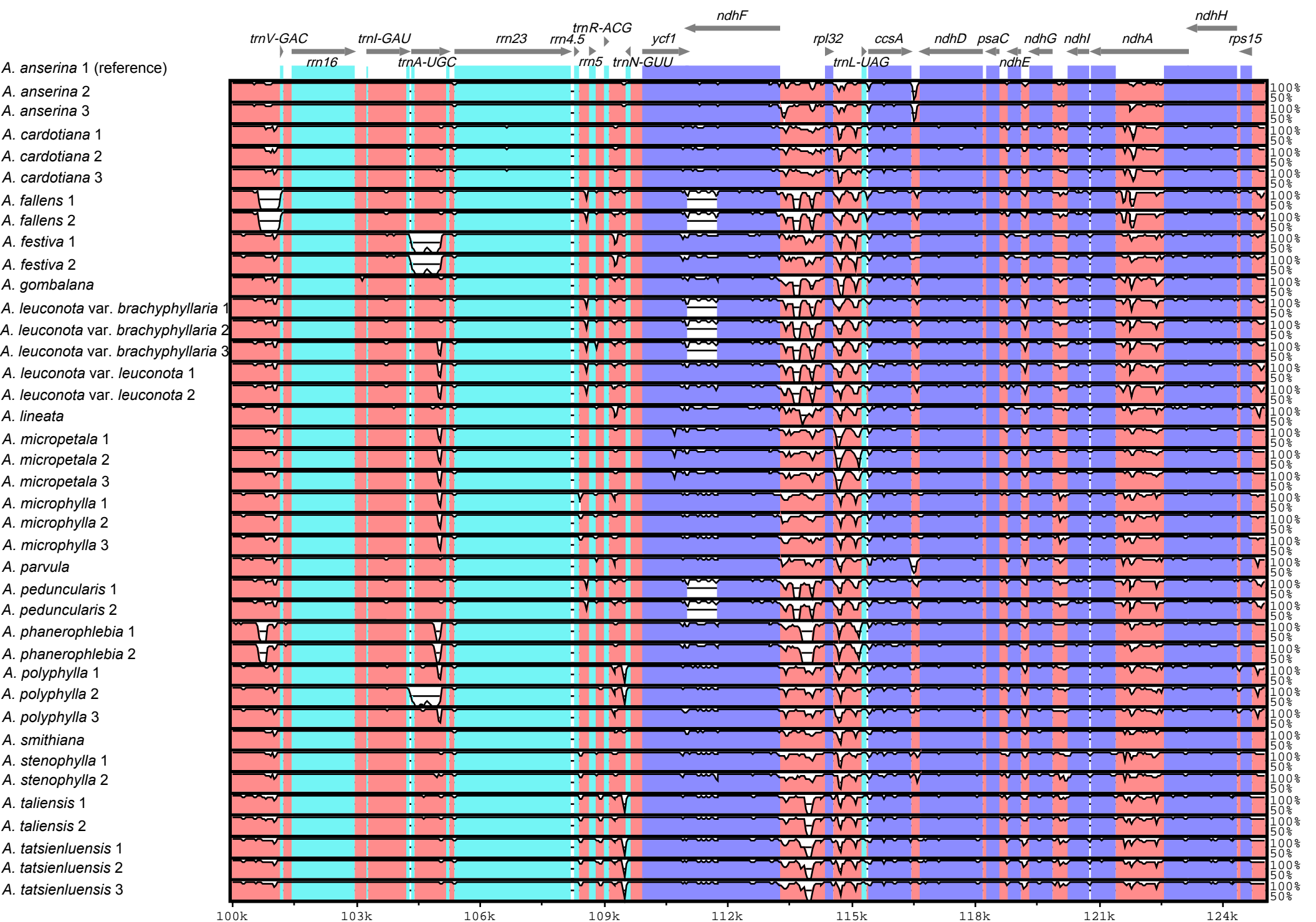

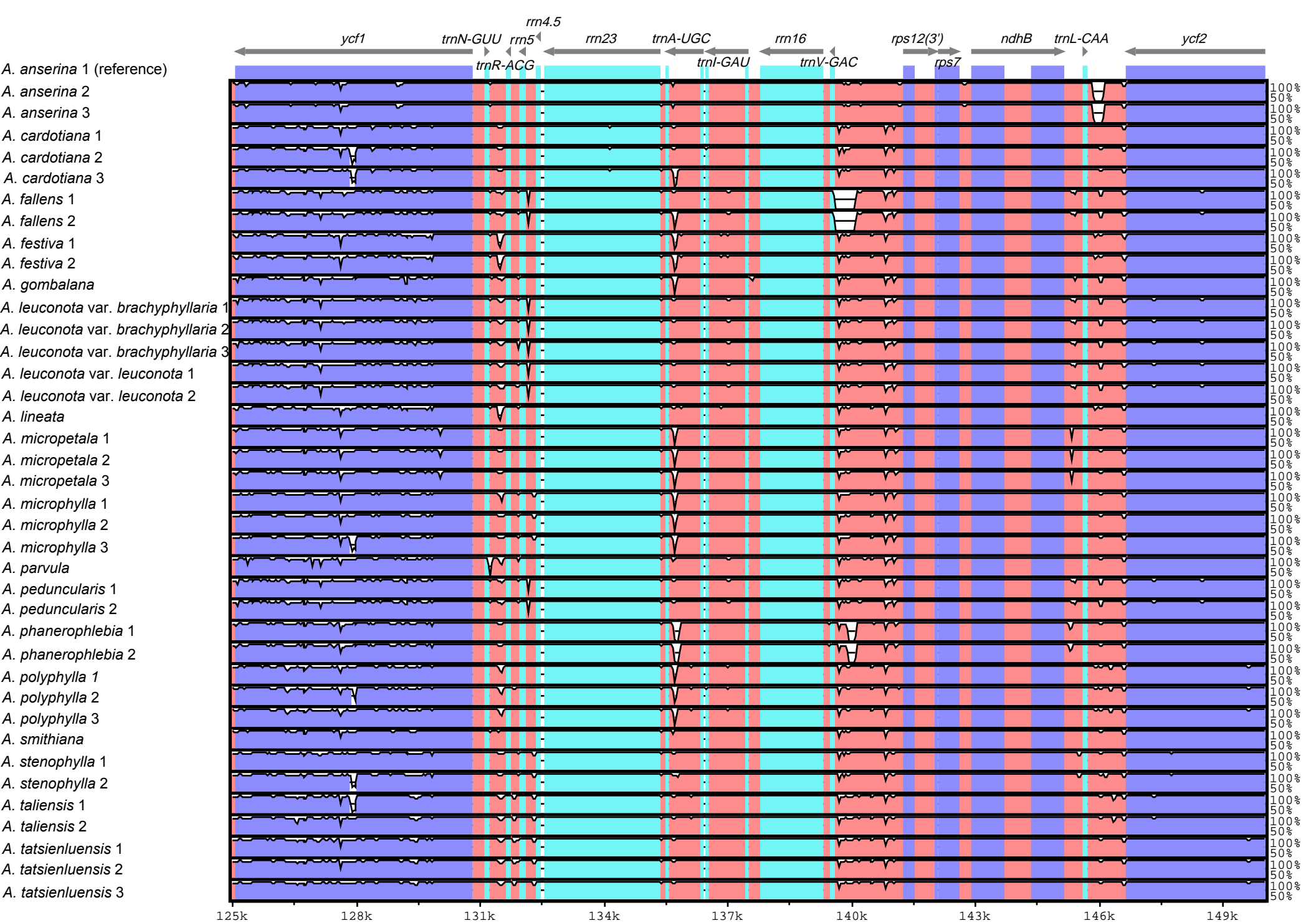

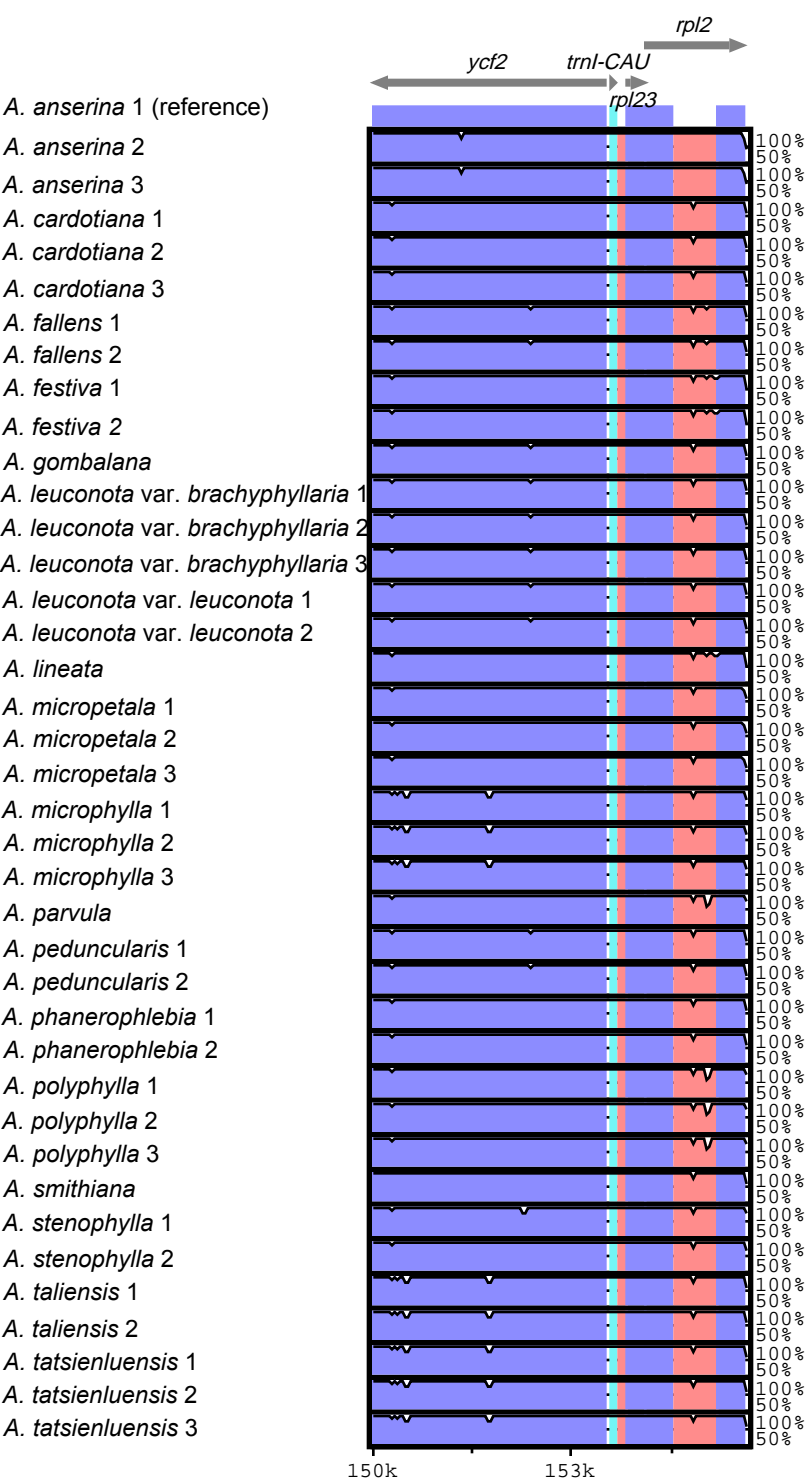

Supplement: Supplementary file 1 [file DataSheet_1.zip › Supplementary Material/Figure S2.pdf]
